# Supplementary material for: Optimized AAV vector enables potent therapeutic rescue of inherited glycosylphosphatidylinositol deficiency in mice
Source: Mol Ther Adv. 2026 Mar 28;34(2):201724. doi: 10.1016/j.omta.2026.201724 (PMC13148894; doi:10.1016/j.omta.2026.201724)
Supplement: Document S1. Figures S1–S7 [file mmc1.pdf]

OMTA, Volume *34*

Supplemental information

Optimized AAV vector enables potent therapeutic rescue of inherited glycosylphosphatidylinositol deficiency in mice

Saori Umeshita, Kae Imanishi, Shibi Likhite, Mika Ito, Naomi Takino, Kathrin C. Meyer, Taroh Kinoshita, Shinichi Muramatsu, and Yoshiko Murakami

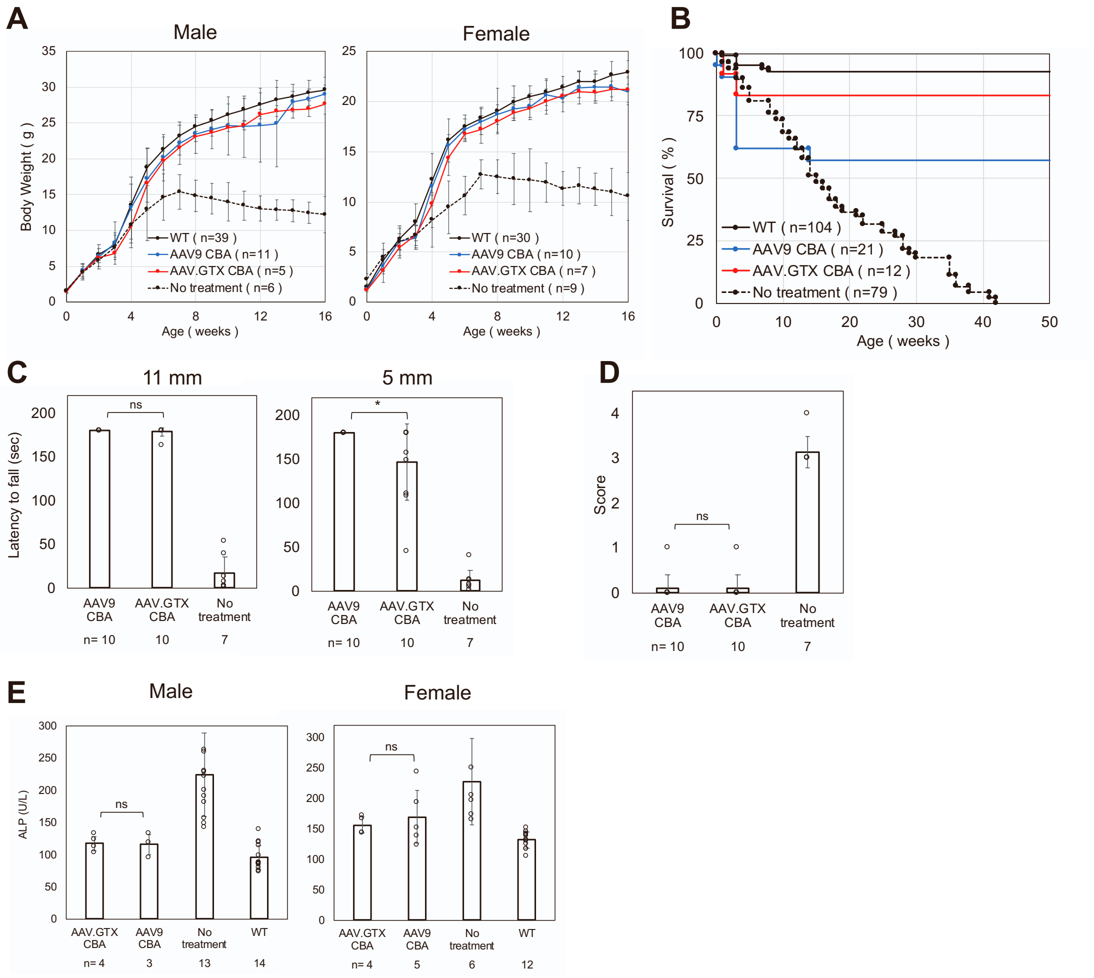

Figure S1. Comparison of AAV9 and AAV.GTX

Intracerebroventricular administration was performed once at $1{\times}10^{11}$ vg/mouse on postnatal days 1–3.

A. Comparison of weekly body weight between AAV9-*CBA-hPIGO* treated KI/KO mice and AAV.GTX-*CBA-hPIGO* treated KI/KO mice. Data are presented as mean ± SD. *n* indicates the number of animals.

B. Kaplan-Meier survival curves for each group. *n* indicates the number of animals.

C. Comparison of latency to fall in the hanging wire test among groups. Data are presented as mean ± SD. *n* indicates the number of animals. (ns: not significant; *p<0.05, Student's two-sided *t*-test) *P*-value for 5mm mesh: AAV9 vs. AAV.GTX, 3.4×10^{-2}.

D. Comparison of tremor score among groups. Data are presented as mean ± SD. *n* indicates the number of animals. (ns: not significant, two-sided *t*-test).

E. Amelioration of hyperphosphatasia. ALP activity was measured in plasma from mice older than 4 months. Data are presented as mean ± SD. *n* indicates the number of animals. (ns: not significant, Student's two-sided *t*-test)

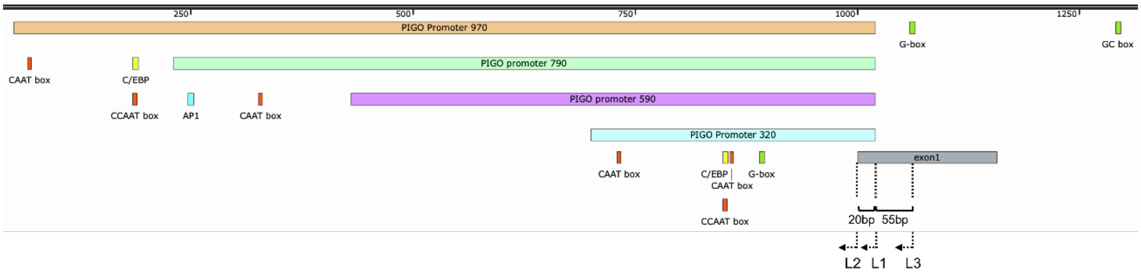

Figure S2. Design of Human Endogenous Promoter of *PIGO* Gene

The transcription factor binding sites were predicted by JASPR, an open-access database. (ref *Nucleic Acids Research*, gkaf1209, https://doi.org/10.1093/nar/gkaf1209)

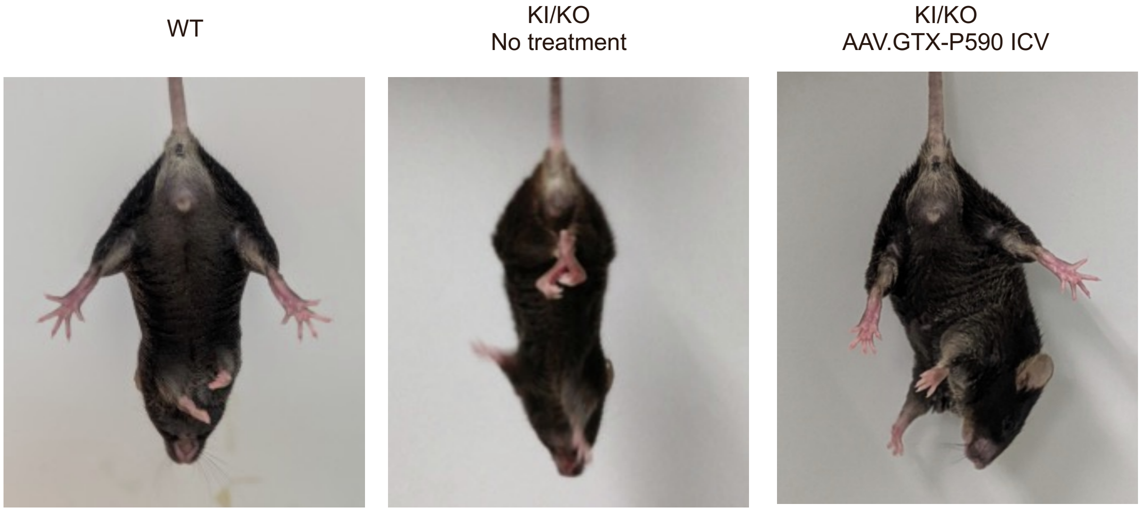

Figure S3. Hindlimb clasping in AAV-treated and untreated KI/KO mice

When mice were suspended by their tail, untreated mice showed hindlimb clasping, while AAV.GTX-*P590* treated mice appeared normal.

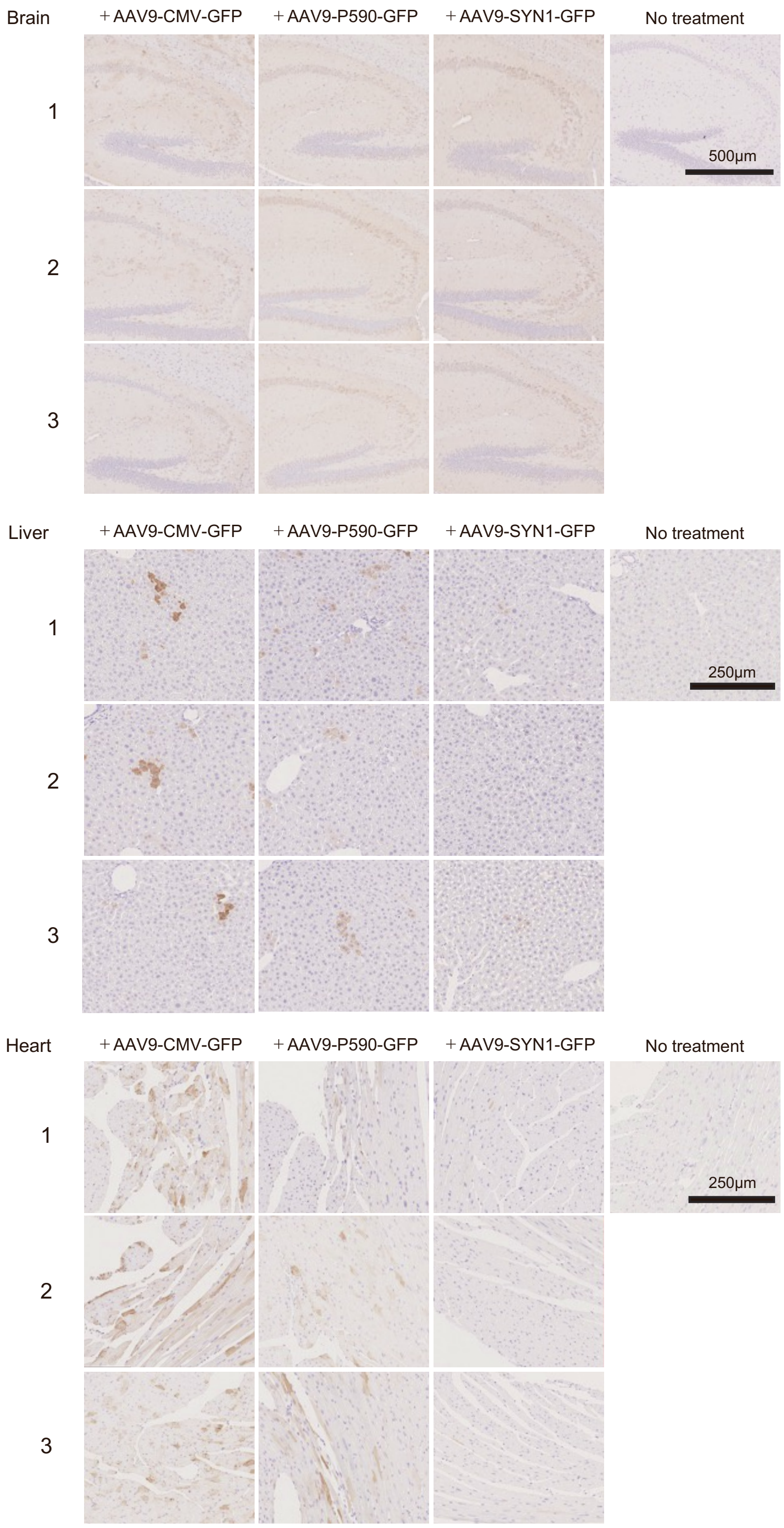

Figure S4. Comparison of GFP DAB staining after ICV injection of AAV9-*GFP* (1 × 10^{11} vg/mouse) driven by various promoters

The results show three cases in each group and one case in the negative control.

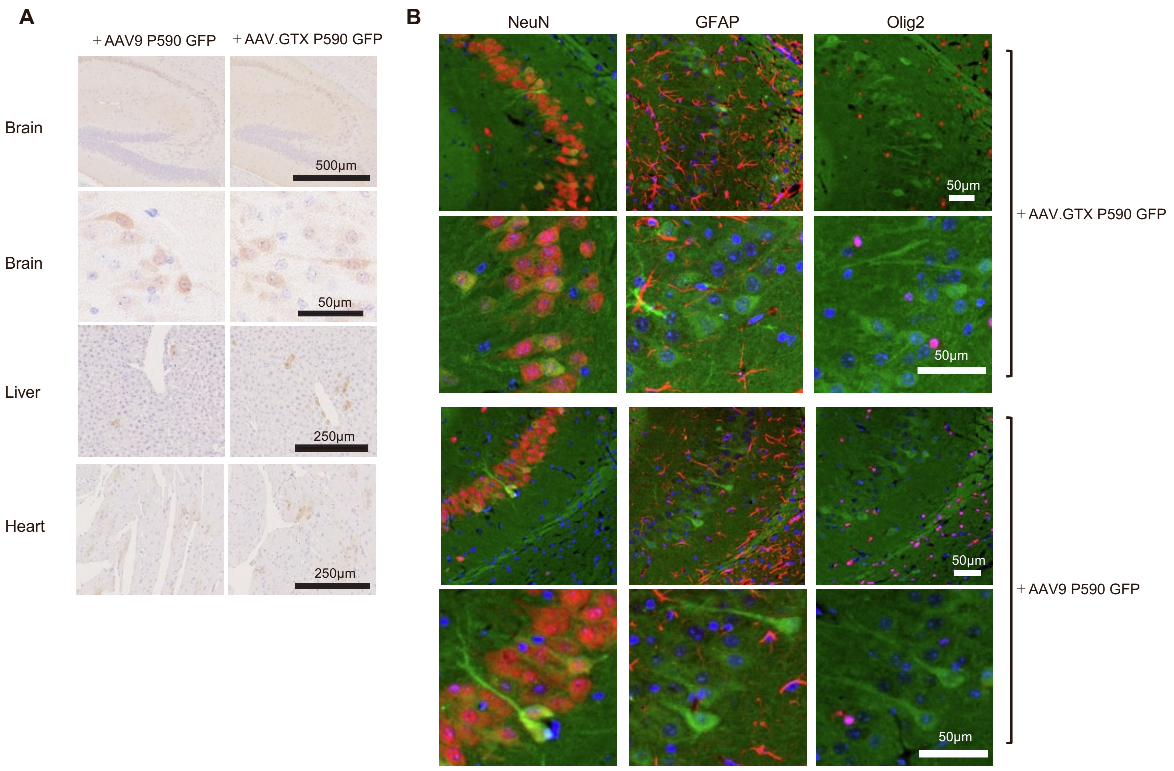

Figure S5. Comparison of GFP tissue staining after ICV injection of AAV.GTX-*P590-GFP* and AAV9-*P590-GFP* (0.3 × 10^{11} vg/mouse)

A. DAB staining for GFP expression.

B. Immunostaining analysis of the mouse hippocampus shown in A. The panels show merged images of GFP (green) and DAPI (blue). Neurons, astrocytes and oligodendrocytes were visualized using Alexa Fluor™ 647-conjugated anti-NeuN, anti-GFAP and anti-Olig2 antibodies, respectively (red).

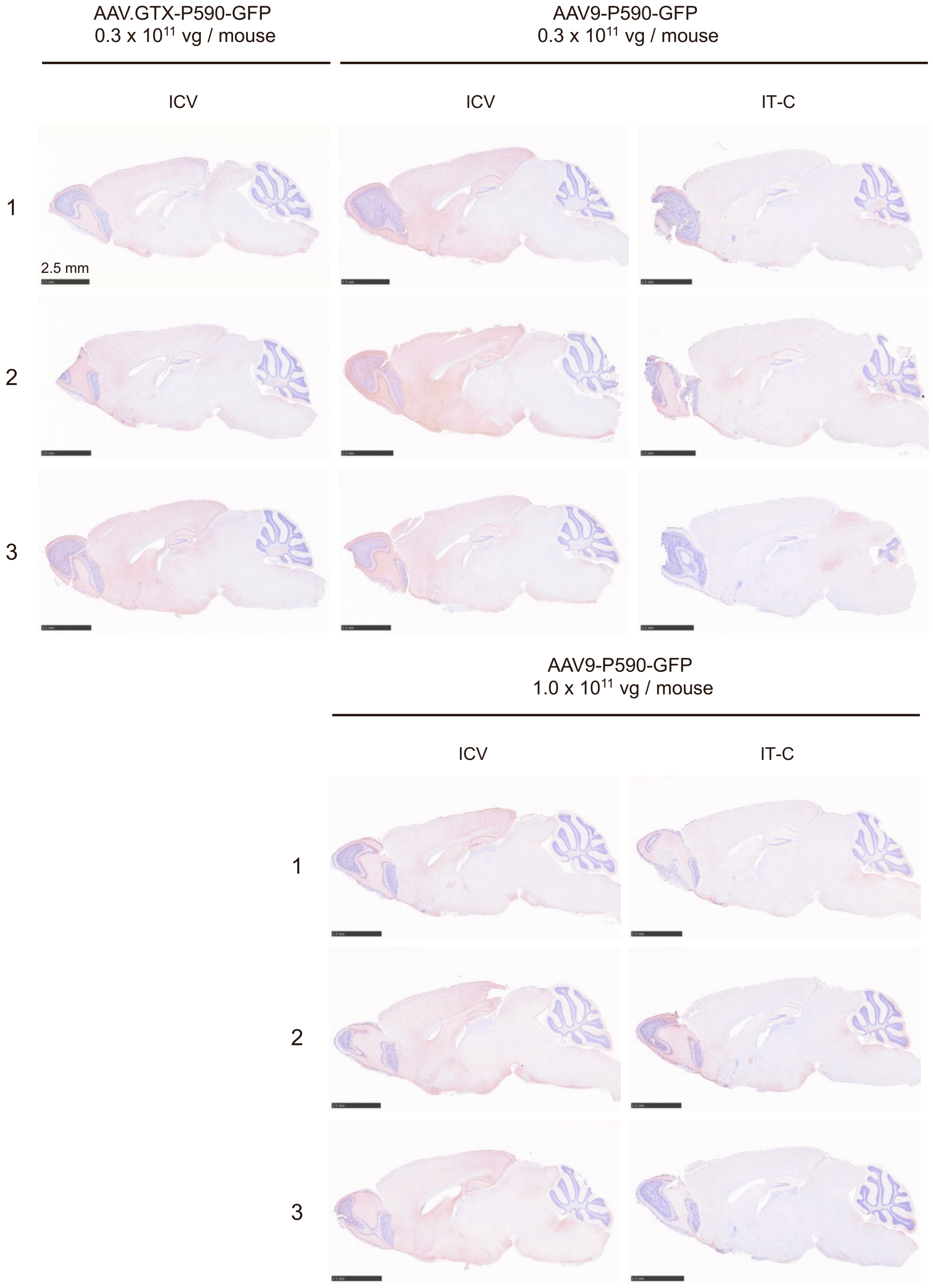

Figure S6. Comparison of DAB staining for GFP in tissue following ICV and IT-C administration of AAV.GTX-*P590-GFP* and AAV9-*P590-GFP* (0.3 or 1.0 × 10^{11} vg/mouse)

The photo shows the whole brain. Results of three cases in each group are shown.

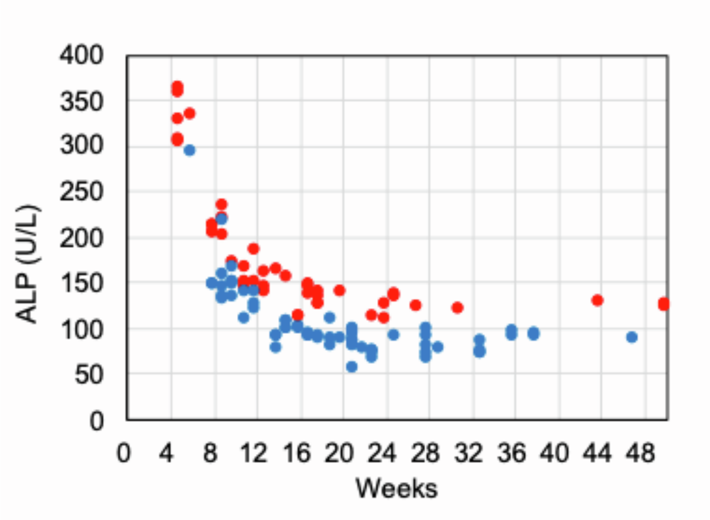

Figure S7. Age-related changes in plasma ALP levels in C57BL/6J mice

Blood samples were collected at various weeks after birth and ALP activity was measured separately for males and females. ALP levels were high in both sexes shortly after birth and gradually decreased by 16 weeks. Females tended to show higher levels than males. Based on these results, we decided to collect blood samples from 16 weeks onwards. Dots represent individual measurements; blue dots indicate males, red dots indicate females.

Video S1. Tremor in AAV-treated and untreated KI/KO mice

Untreated mice showed severe tremor, while AAV-treated mice appeared normal. Single black line on the tail indicates an AAV-treated mouse, two lines indicate an untreated mouse, and no line indicates a wild-type mouse.
