# Supplementary material for: Optimized AAV vector enables potent therapeutic rescue of inherited glycosylphosphatidylinositol deficiency in mice
Source: Mol Ther Adv. 2026 Mar 28;34(2):201724. doi: 10.1016/j.omta.2026.201724 (PMC13148894; doi:10.1016/j.omta.2026.201724)
Supplement: Document S2. Article plus supplemental information [file mmc3.pdf]

Molecular Therapy
Advances
Original Article

Optimized AAV vector enables potent therapeutic rescue of inherited glycosylphosphatidylinositol deficiency in mice

Saori Umeshita,[1] Kae Imanishi,[1] Shibi Likhite,[2] Mika Ito,[3] Naomi Takino,[3] Kathrin C. Meyer,[2,4] Taroh Kinoshita,[1,5] Shinichi Muramatsu,[3] and Yoshiko Murakami[1]

[1]Laboratory of Immunoglycobiology, Research Institute for Microbial Diseases, Osaka University, Suita, Osaka 565-0871, Japan; [2]Center for Gene Therapy, Abigail Wexner Research Institute, Nationwide Children's Hospital, Columbus, OH 43215, USA; [3]Division of Neurological Gene Therapy, Jichi Medical University Shimotsuke, Osaka 329-0498, Japan; [4]Department of Pediatrics, The Ohio State University, Columbus, OH 43210, USA; [5]Center for Infectious Disease Education and Research, Osaka University, Suita, Osaka 565-0871, Japan

Thirty genes are involved in the biosynthesis and modification of glycosylphosphatidylinositol (GPI)-anchored proteins. Defects in these genes cause inherited GPI deficiency, whose main clinical features include intellectual disability, developmental delay, and intractable seizures, and sometimes hyperphosphatasia and multiple congenital anomalies. The mechanisms of its systemic, especially neurological, manifestations largely remain unclear, and no fundamental therapy has yet been established. In this study, we report the development of effective adeno-associated virus (AAV)-mediated gene therapy using a mouse model of inherited GPI deficiency caused by phosphatidylinositol glycan anchor biosynthesis class O (PIGO) mutations, with the aim of developing gene therapy applicable to human patients. By optimizing AAV vectors with the most effective and safest promoter, we achieved significant amelioration of neuronal phenotype and growth impairment, with no cases of liver cancer observed. We further determined the optimal administration route and therapeutic dose required for effective systemic delivery. These results provide proof of concept for the therapeutic efficacy of AAV-based gene replacement therapy for inherited GPI deficiency.

INTRODUCTION

Glycosylphosphatidylinositol (GPI) anchor is a glycolipid that attaches approximately 160 proteins to the plasma membrane of mammalian cells. These GPI-anchored proteins (GPI-APs) play important roles in embryogenesis, neurological development, immune system, and fertility; therefore, complete loss of GPI biosynthesis causes embryonical lethality. Thirty genes are involved in the biosynthesis and transport of GPI-APs, and hypomorphic mutations in these genes cause inherited GPI deficiency (IGD).[1]

We reported the first IGD case—PIGM (phosphatidylinositol glycan anchor biosynthesis, class M) deficiency—in collaboration with a group in England.[2,3] Since then, many forms of IGD have been identified due to advances in next-generation sequencing. To date, 24 types of IGD have been reported, presenting with intellectual disability, intractable seizures, and sometimes hyperphosphatasia, digital anomalies, or multi-organ abnormalities.[4] Approximately 70 cases have been identified in Japan and about 700 cases worldwide.[4]

High-dose administration of the vitamin B6 derivative pyridoxine is known to be effective in controlling seizures in some patients.[5] Alkaline phosphatase (ALP) is a GPI-AP whose cell-surface expression is reduced in IGD patients. Cell-surface ALP converts pyridoxal phosphate, the active form of vitamin B6, to pyridoxal, which can be taken up into cells and re-phosphorylated. Pyridoxal phosphate serves as a cofactor for many enzymes, including glutamate decarboxylase, which synthesizes Gamma-Amino Butyric Acid (GABA). Reduced GABA production is thought to contribute to the intractable seizures observed in IGD.[6,7] A folate receptor is also a GPI-AP, and its decreased expression is considered another cause of seizures and cerebellar dysfunction[8]; therefore, supplementation with folinic acid has been attempted.[9] However, there is currently no fundamental treatment for IGD.

We generated a mouse model of PIGO deficiency (PIGO-IGD), which has a relatively higher number of reported cases in Japan, by knocking in patient-derived mutations.[10] This model faithfully recapitulates patient symptoms, including reduced GPI-AP expression on granulocytes, growth retardation, spontaneous seizures, hyperphosphatasia, impaired muscle strength and coordination, and shortened lifespan. *PIGO* (*phosphatidylinositol glycan anchor biosynthesis class O*) gene encodes for the enzyme that attaches ethanolamine phosphate to the third mannose residue of GPI.[11]

We first attempted genome editing using the homologyindependent targeted integration assisted with a low level of transgene expression

Received 5 January 2026; accepted 25 March 2026;
https://doi.org/10.1016/j.omta.2026.201724.

Correspondence: Yoshiko Murakami, Research Institute for Microbial Diseases, Osaka University, 3-1 Yamada-oka, Suita, Osaka 565-0871, Japan.
E-mail: yoshiko@biken.osaka-u.ac.jp

© 2026 The Author(s).
Published by Elsevier Inc. on behalf of The American Society of Gene and Cell Therapy.
This is an open access article under the CC BY license (http://creativecommons.org/licenses/by/4.0/).

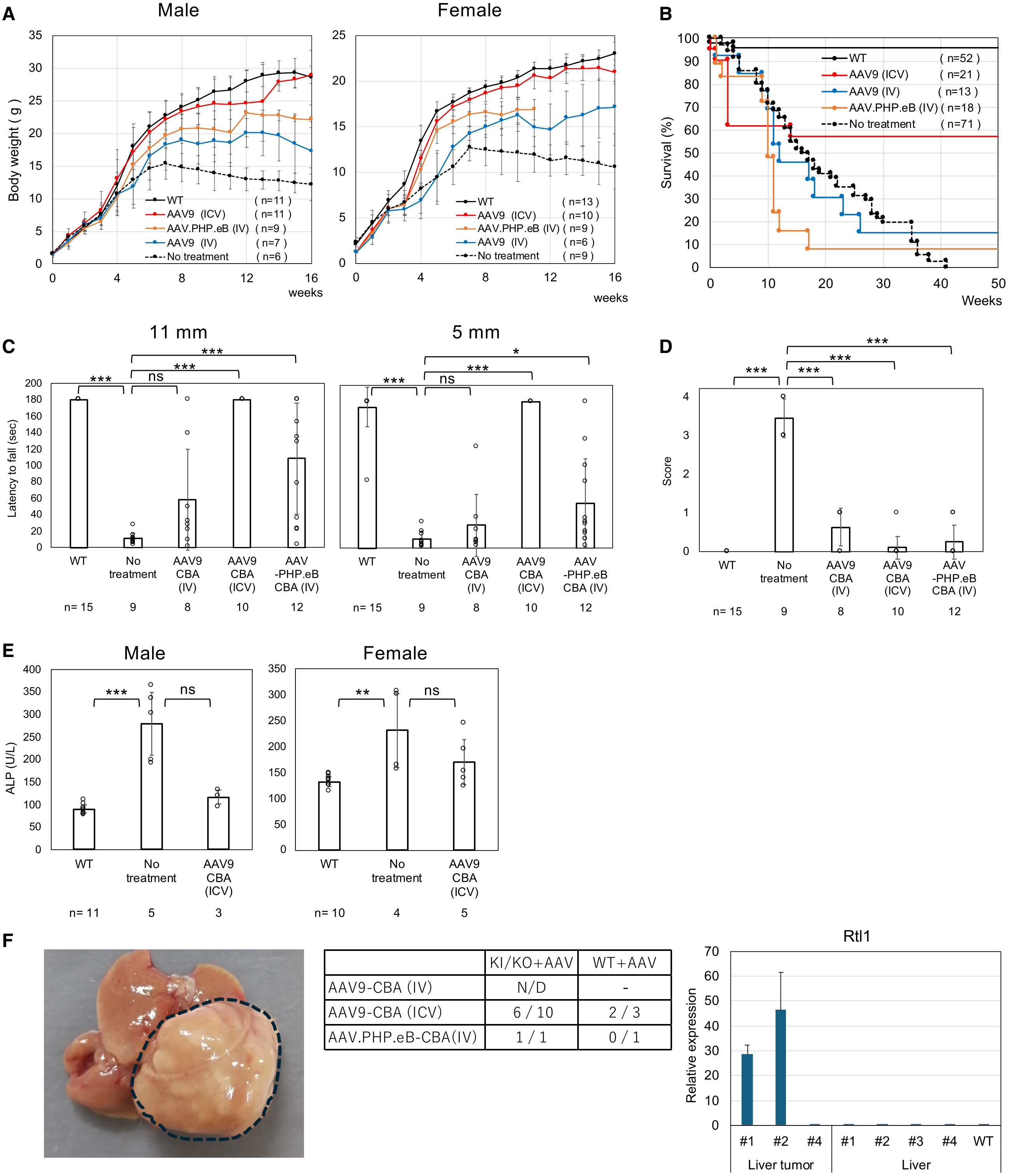

	KI/KO+AAV	WT+AAV
AAV9-CBA (IV)	N/D	-
AAV9-CBA (ICV)	6 / 10	2 / 3
AAV.PHP.eB-CBA(IV)	1 / 1	0 / 1

Figure 1. Administration of AAV-*CBA-hPIGO* (1 × 10^{11} vg/mouse) was effective but hepatocellular carcinoma developed

(A) Comparison of weekly body weight in AAV-treated KI/KO mice and untreated KI/KO mice. Data are presented as mean ± SD. *n* indicates the number of animals. (B) Kaplan-Meier survival curves for each group. *n* indicates the number of animals. (C) Comparison of the latency to fall in the hanging wire test among groups. Data are presented as mean ± SD. *n* indicates the number of animals. Statistical analysis was performed using one-way ANOVA followed by Dunnett's post hoc test (*p < 0.05;

(legend continued on next page)

(HITI-TE) method delivered by adeno-associated virus (AAV) to treat the model mice. The treatment was highly effective, and all phenotypes were reversible when administered during the neonatal stage.[10] To develop therapeutic strategies for IGD patients, we next treated nervous-system-specific *Piga*-knockout (KO) mice (the PIGA-IGD model) using genome-supplementation therapy via intravenous administration of AAV.PHP.eB, demonstrating its therapeutic efficacy.[12]

Here, with the aim of developing gene therapy applicable to human patients, we optimized an AAV vector using the most effective and safest promoter and determined the optimal administration route and dosage using the PIGO-IGD mouse model, thereby establishing proof of concept (POC) for therapeutic efficacy.

RESULTS

Administration of AAV-*CBA-hPIGO* was effective, but hepatocellular carcinoma developed

Previously, we generated PIGO-IGD model mice by knocking in the patient-derived Thr130Arg mutation.[10] Compound heterozygous mice (KI/KO), obtained by mating heterozygous knockin (KI) mice with heterozygous KO mice, faithfully recapitulated patient symptoms.[10] Because Zolgensma, which is clinically used for spinal muscular atrophy, employs AAV9 with *the chicken β-actin* (*CBA*) promoter, we attempted neonatal treatment of KI/KO mice with AAV9-*CBA-hPIGO*, comparing its therapeutic efficacy with AAV.PHP.eB-*CBA-hPIGO* administered intravenously at 1.0×10^{11} vg/mouse. In contrast to AAV.PHP.eB, which efficiently crosses the Blood-Brain Barrier (BBB) but is not applicable to humans,[13,14] AAV9 crosses the BBB inefficiently (Figures 1A–1E). As expected, treatment with AAV9-*CBA-hPIGO* showed insufficient efficacy, prompting us to switch to intracerebroventricular (ICV) administration.

Body weights of untreated and treated mice were monitored weekly until 16 weeks of age (Figure 1A). ICV administration of AAV9-*CBA-hPIGO* ameliorated growth impairment in both male and female mice. Furthermore, whereas untreated mice died by 40 weeks of age, survival was significantly improved in the treated group (Figure 1B). Hanging-test performance was clearly improved in AAV-treated KI/KO mice, indicating amelioration of muscle weakness and impaired motor coordination (Figure 1C). Tremor severity was also markedly reduced in treated mice (Figure 1D). At 4 months of age, plasma ALP levels were decreased in the treated male mice (Figure 1E). These findings indicate that AAV administration successfully supplemented GPI biosynthesis.

Thus, AAV-based gene replacement therapy appeared promising for curative treatment of IGD. However, upon necropsy 1 year after administration, hepatocellular carcinomas were detected at high frequency (Figure 1F).

We previously reported that treatment of *Nestin-Cre/Piga-floxed* mice with AAV.PHP.eB-*CBA-hPIGA* similarly resulted in liver cancer.[12] Liver tumors are known to frequently develop more than 1 year after neonatal AAV administration when strong promoters, such as CBA or the liver-specific thyroxine-binding globulin (TBG) promoter, are used.[15] Although AAV is generally considered non-integrating, neonatal administration can result in frequent integration into the Rian locus—a non coding RNA (ncRNA) cluster—in hepatocytes, leading to activation of the adjacent oncogene*retrotransposon-like 1* (*Rtl1*) and subsequent tumor formation.[15] We analyzed *Rtl1* expression in normal-appearing liver tissue and liver tumors from AAV-treated mice by quantitative reverse-transcription PCR (RT-qPCR). In two of three tumor tissues, *Rtl1* expression was markedly elevated (Figure 1F), suggesting AAV integration into the Rian locus. Although this phenomenon is currently thought to be mouse specific, it underscores the need to select a promoter with lower toxicity.

AAV.GTX is an improved version of AAV9

In AAV.GTX, two tyrosine residues on the capsid surface (Y446 and Y731) are substituted with phenylalanine (Y446F and Y731F).[16] We previously showed that AAV.GTX enables more efficient gene transfer and greater intracellular stability than AAV9.[16] This vector is already in clinical use for other gene therapies.[17] We compared AAV.GTX-*CBA-hPIGO* with AAV9-*CBA-hPIGO* and found that both vectors were similarly effective in alleviating symptoms (Figures S1A–S1E).

P590L1 was selected as the endogenous hPIGO promoter for gene therapy

We selected endogenous promoter regions of various lengths from the human *PIGO* gene (Figure S2) and evaluated their activities using luciferase reporter assays in HEK293 cells and in Neuro2a cells, a mouse neuroblastoma cell line. The results showed that the L1 promoter fragment exhibited the strongest activity (Figure 2A) and that the four endogenous promoters displayed similar activity levels,

$p < 0.001$; ns, not significant). For the 11 mm mesh (ANOVA $p = 2.8 \times 10^{-13}$), wild type and AAV9-*CBA* (ICV) and AAV.PHP.eB differed from no treat (all $p < 0.001$), whereas AAV9-*CBA* (IV) did not ($p = 0.143$). For the 5 mm mesh (ANOVA $p = 2.2 \times 10^{-18}$), wild type and AAV9-*CBA* (ICV) differed from no treat ($p < 0.001$), AAV.PHP.eB showed a modest effect ($p = 0.03$), and AAV9-*CBA* (IV) did not. (D) Comparison of tremor score among groups. Scores range from 0 to 4 (severest). Data are presented as mean ± SD. *n* indicates the number of animals. Statistical analysis was performed using one-way ANOVA followed by Dunnett's post hoc test ($p < 0.001$). ANOVA $p = 5.8 \times 10^{-26}$; all groups differed significantly from no treat (all $p < 0.001$). (E) Amelioration of hyperphosphatasia. ALP activity was measured in plasma from mice older than 4 months. Data are presented as mean ± SD. *n* indicates the number of animals. Statistical analysis was performed using the Kruskal-Wallis test followed by Dunn's post hoc test (**$p < 0.01$; ***$p < 0.001$; ns, not significant). Males: overall $p = 1.3 \times 10^{-3}$; wild type differed from no treat ($p < 0.001$), whereas AAV9-*CBA* (ICV) did not ($p = 0.26$). For females, overall $p = 0.011$; wild type differed from no treat ($p = 0.003$), whereas AAV9-*CBA* (ICV) did not ($p = 0.18$). (F) Left: photographs of liver tumors in a 1-year-old mouse. Middle: table showing the number of confirmed liver cancer cases. Right: relative expression of *Rtl1* in tumors and in normal liver tissue. Data are presented as mean ± SD.

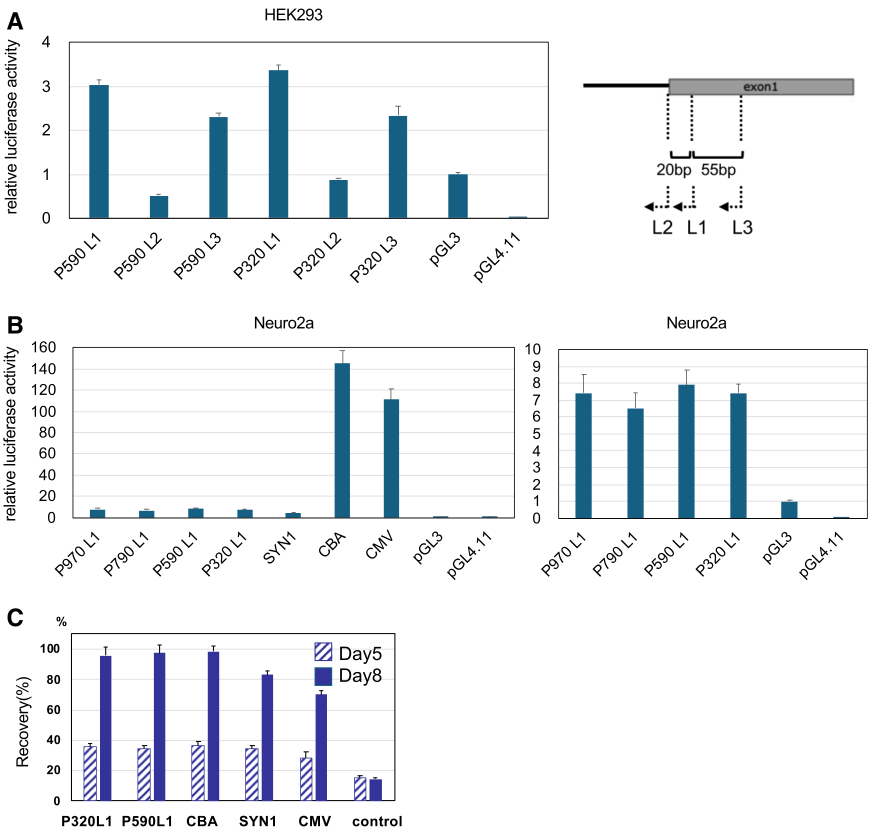

Figure 2. Design of endogenous PIGO promotor

Several endogenous promoters of the *PIGO* gene were cloned and their activities were using a luciferase reporter assay. (A) Measurement of endogenous *PIGO* promoter activity in HEK293 (luciferase activity). Luciferase activity of pGL3 (SV40 promoter) was set to 1 for comparison. Activities of L1, L2, and L3 promoters were compared. L2 showed loss of activity, whereas L1 exhibited the highest activity. (B) Measurement of endogenous *PIGO* promoter activity in Neuro2a cells (luciferase activity). Luciferase activity of pGL3 is set to 1 for comparison. No significant difference in activity was observed among the promoter lengths. (C) Percentage of recovery in CD24 expression after AAV infection in Neuro2a cells. The endogenous *PIGO* promoter restored CD24 expression to a level comparable to that achieved with the *CBA* promotor.

although they were much weaker than strong viral promoters, such as *CBA* or *CMV* (Figure 2B).

Considering the packaging capacity of AAV, we selected *P590L1* and *P320L1*, generated AAV.GTX vectors containing these promoters, and infected Pigo-deficient Neuro2a cells. Restoration of CD24, a GPI-AP, measured by flow cytometry, was comparable to that achieved with AAV.GTX-*CBA-PIGO* (Figure 2C), indicating that even a weak promoter was sufficient to drive *PIGO* expression. Based on these results, *P590L1* was chosen for subsequent animal experiments and is referred to as *P590* hereafter.

P590 promoter was the most effective among various promoters

We next compared the effectiveness of AAV.GTX vectors expressing *hPIGO* under different promoters—including the *P590* promoter, a *Synapsin1* (*SYN1*) promoter, the *CMV* promoter, and the *CBA* promoter—in treating the model mice. ICV administration was performed once at 1×10^{11} vg/mouse on postnatal days 1–3. All promoters produced favorable therapeutic effects compared with untreated controls, including improved weight gain (Figure 3A) and survival (Figure 3B), enhanced muscle strength and coordination (Figure 3C), reduced tremors (Figure 3D), reduced Pentylenetetrazol (PTZ)-induced seizures (Figure 3E), and significantly decreased ALP levels in males but not in females, possibly due to the small sample size (Figure 3F). Among these, the *P590* promoter yielded the greatest improvement across all parameters. All untreated KI/KO mice exhibited hindlimb clasping, whereas *P590* promotor-treated mice did not, and tremors were markedly milder compared to untreated controls (Figure S3 and Video S1).

When liver tumor formation was examined 1 year after administration, only a single case—associated with the *CBA* promoter—was observed. No liver cancers were detected in mice treated with any of the other promoters (Figure 3G).

hPIGO expression in tissues of mice treated with AAV vectors carrying various promoters

We measured *hPIGO* expression in various tissues of treated mice 1 year after AAV.GTX administration using qPCR. Although *Pigo* KI/KO condition might affect distribution and/or duration of *hPIGO* expression, we had to use wild-type mice in this study solely because of the availability of sufficient numbers of KI/KO newborns. *hPIGO* driven by the *CBA*, *P590*, and *SYN1* promoters was expressed predominantly in the cerebrum, whereas *hPIGO* driven by the *CMV* promoter exhibited high expression in muscle tissue (Figure 4A). As expected, the *hPIGO* expression driven by the *SYN1* promoter was restricted to the brain. When comparing *hPIGO* expression levels across tissues for each promoter, we found that *P590* showed lower expression than *CBA* in all tissues that were examined (Figure 4B).

We also analyzed the tissue distribution of AAV9 vectors with different promoters by immunostaining various tissues from treated mice. To do this, we generated AAVs expressing GFP under each promoter and administered them via ICV to wild-type newborn mice. The mice were sacrificed 4 months after administration, and organs—including the brain, heart, and liver—were collected for

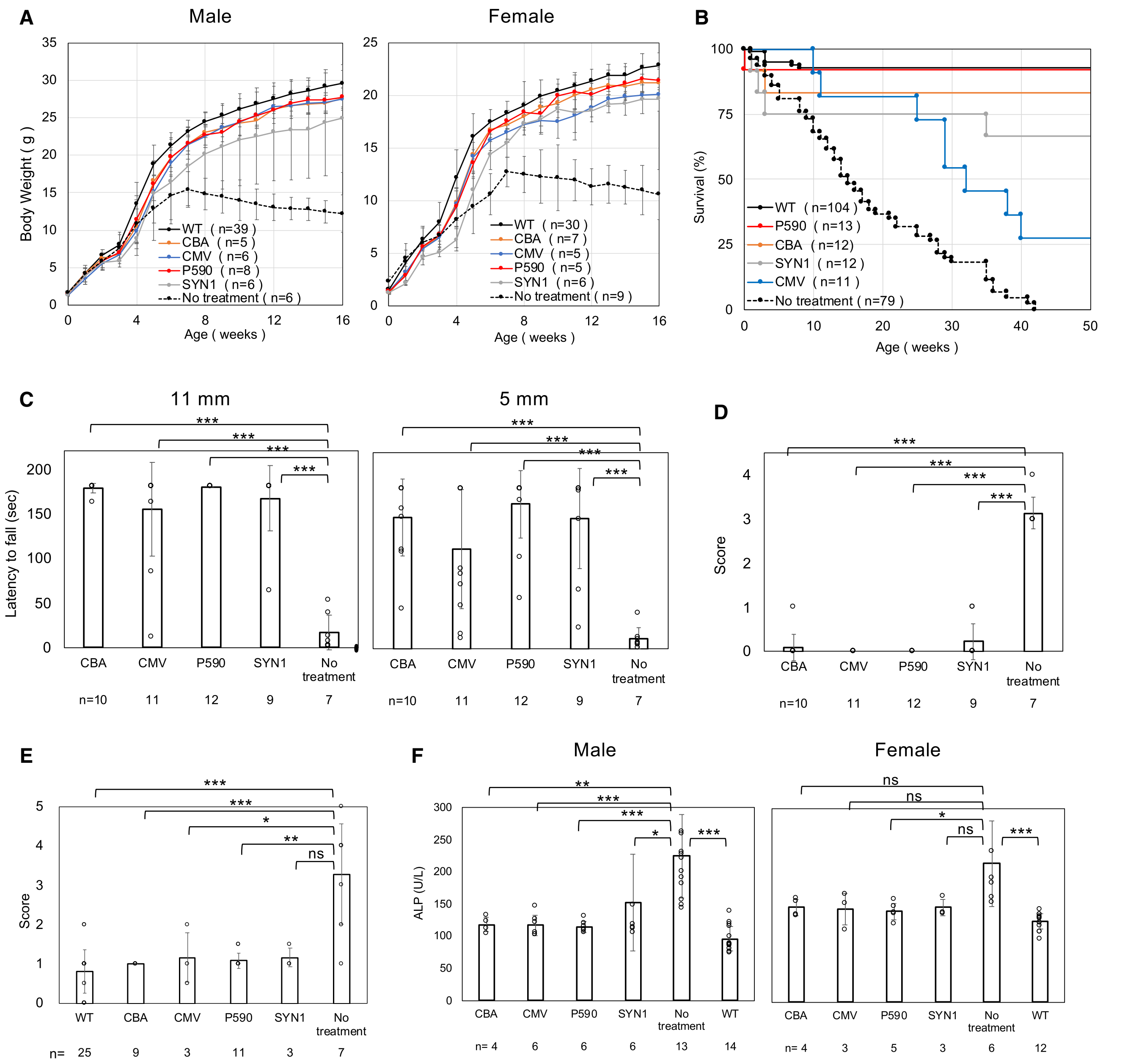

G

	KI/KO+AAV	WT+AAV
AAV.GTX-CBA (ICV)	0 / 9	1 / 3
AAV.GTX-CMV (ICV)	0 / 3	0 / 7
AAV.GTX-P590 (ICV)	0 / 9	0 / 4
AAV.GTX-SYN1 (ICV)	0 / 8	0 / 2

Figure 3. Comparative evaluation of the *in vivo* effectiveness of AAV.GTX vectors with various promoters

Among the promoters tested, *P590* was the most effective in reversing symptoms in mice. (A) Comparison of weekly body weight in AAV-treated KI/KO mice and untreated KI/KO mice. Data are presented as mean ± SD. *n* indicates the number of animals. (B) Kaplan-Meier survival curves for each group. *n* indicates the number of animals. (C) Comparison of the latency to fall in the hanging wire test among groups. Data are presented as mean ± SD. *n* indicates the number of animals. Statistical analysis was

(legend continued on next page)

3,3′ Diaminobenzidine (DAB) staining to detect GFP with anti-GFP antibody. Consistent with the qPCR results, GFP driven by the *SYN1* promoter was expressed exclusively in the brain, whereas GFP driven by the *CMV* promoter was strongly expressed in the liver and heart as well as in the brain, though with distinct expression patterns. GFP driven by the *P590* promoter was expressed primarily in the brain, with low-level staining observed in both liver and heart (Figure 4C). These results are representative of analyses performed on three mice per group. The data including the other two mice are shown in the supplemental information (Figure S4).

We further performed double staining using neuron-, astrocyte-, or oligodendrocyte-specific markers together with GFP and found that GFP expression driven by the *P590* promoter was localized specifically to neurons (Figure 4D). AAV.GTX-*GFP* showed a GFP-expression pattern comparable to that of AAV9, with expression predominantly restricted to neurons (Figures S5A and S5B).

Optimization of AAV.GTX-*P590-hPIGO* delivery for therapeutic application in IGD

To advance toward clinical application, we attempted to optimize the dosage using AAV.GTX-*P590-hPIGO* (Figures 5A–5E). ICV administration was performed once at doses of 1×10^{11}, 3×10^{10}, or 1×10^{10} vg/mouse on postnatal days 1–3. As the dosage decreased, the therapeutic efficacy declined.

Because clinical treatment in humans is expected to use intrathecal administration, we evaluated delivery through the cisterna magna (intracisternal, IT-C) and confirmed that it provided therapeutic effects comparable to those of ICV administration (Figures 6A–6D). We also administered AAV.GTX-*P590-GFP* via IT-C and compared the distribution of GFP expression in the brain with that obtained by ICV administration. GFP delivered via IT-C was expressed in neurons of the cerebral cortex and in cerebellar Purkinje cells, whereas ICV administration resulted in strong neuronal expression in the hippocampus in addition to the cerebral cortex and cerebellar Purkinje cells (Figure 6E). These results are representative of analyses performed on three mice per group. The data including the other two mice are shown in the supplemental information (Figure S6).

We also treated 4-week-old mice with AAV9-*P590-hPIGO*. A dose of 1×10^{11} vg/mouse was ineffective, but increasing the dose 10-fold resulted in significant improvements in growth, muscle strength and coordination, and tremors, although the effect was not as pronounced as in newborn-treated mice (Figures 7A–7D). These results suggest that the therapy can also be effective in older children.

DISCUSSION

More than 160 types of GPI-APs are expressed on mammalian cells, and their expression is variably decreased in IGD patients. Because GPI-APs play essential roles throughout fetal development and complete loss of GPI biosynthesis results in early embryonic lethality,[18] we were surprised to find that gene therapy initiated during the neonatal period could almost completely normalize the phenotype, at least with respect to motor development.

Previously, we established PIGO-deficient model mice that closely recapitulate the patients' symptoms and treated them using HITI-TE, a genome-editing method delivered by intravenous injection of AAV.PHP.eB.[10] This approach was highly effective in reversing the phenotype. To further develop a clinically applicable gene therapy, we next aimed to identify the optimal serotype and promoter, the safest route of administration, and the minimum effective dose.

Regarding the serotype, AAV9 is commonly used for supplemental gene therapy targeting neurological disorders.[19] Unlike AAV.PHP.eB—which can be used only in C57BL/6 mice and is capable of crossing the BBB—AAV9 does not efficiently cross the BBB[13,14]; therefore, we switched from intravenous to ICV administration. The AAV capsid plays a critical role in determining cellular tropism, the rate of cellular entry, and the efficiency of transgene expression. There is active development going on to modify the amino acid sequence of the capsid so that it can reach target organs but not the liver.[20,21] Thus, rational engineering of capsid properties is considered an important strategy to improve both efficacy and safety in gene therapy. Tyrosine residues on the AAV capsid are phosphorylated and subsequently ubiquitinated, marking the vector for proteasomal degradation before it can reach the nucleus.[22]We demonstrated that AAV.GTX, an AAV9-derived capsid harboring the combined Y446F and Y731F substitutions, reduces phosphorylation of these residues, thereby allowing the vector to evade proteasomal degradation, enhance nuclear delivery, and increase transgene

performed using one-way ANOVA followed by Dunnett's post hoc test (***$p < 0.001$). For the 11 mm mesh (ANOVA $p = 3.3 \times 10^{-13}$) and 5 mm mesh (ANOVA $p = 2.2 \times 10^{-6}$), all treatment groups (*CBA*, *CMV*, *P590*, and *SYN1*) differed significantly from the no treat group (*CBA*, *P590*, and *SYN1*) $p < 0.001$. (D) Comparison of tremor score among groups. Data are presented as mean ± SD. *n* indicates the number of animals. Statistical analysis was performed using one-way ANOVA followed by Dunnett's post hoc test (***$p < 0.001$). ANOVA $p = 4.2 \times 10^{-27}$; all treatment groups (*CBA*, *CMV*, *P590*, and *SYN1*) differed significantly from the no treat group (all $p < 0.001$). (E) Evaluation of PTZ-induced seizures. Mice were injected intraperitoneally with the dose of 20 mg/kg PTZ. Data are presented as mean ± SD. *n* indicates the number of animals. Statistical analysis was performed using the Kruskal-Wallis test followed by Dunn's post hoc test (*$p < 0.05$; **$p < 0.01$; ***$p < 0.001$). Overall $p = 4.6 \times 10^{-4}$; wild type, *CBA*, *CMV*, and *P590* differed from no treat ($p < 0.001$, $p < 0.001$, $p = 0.023$, and $p = 0.0037$, respectively), whereas *SYN1* did not ($p = 0.086$). (F) Amelioration of hyperphosphatasia. ALP activity was measured in plasma from mice older than 4 months. Data are presented as mean ± SD. *n* indicates the number of animals. For males, statistical analysis was performed using one-way ANOVA followed by Dunnett's post hoc test (*$p < 0.05$; **$p < 0.01$; ***$p < 0.001$). Overall $p = 8.3 \times 10^{-6}$; all groups (*CBA*, *CMV*, *P590*, *SYN1*, and wild type) differed from no treat ($p = 1.5 \times 10^{-3}$, 1.9×10^{-4}, 1.9×10^{-4}, 0.017, and $p < 0.0001$, respectively). For female, statistical analysis was performed using the Kruskal-Wallis test followed by Dunn's post hoc test (*$p < 0.05$; ***$p < 0.001$; ns, not significant). Overall $p = 1.7 \times 10^{-3}$; P590 and wild type differed from no treat ($p = 0.038$ and $p < 0.001$, respectively), whereas *CBA*, *CMV*, and *SYN1* did not ($p = 0.09$, 0.11, and 0.24, respectively). (G) The table shows the number of confirmed liver cancer cases.

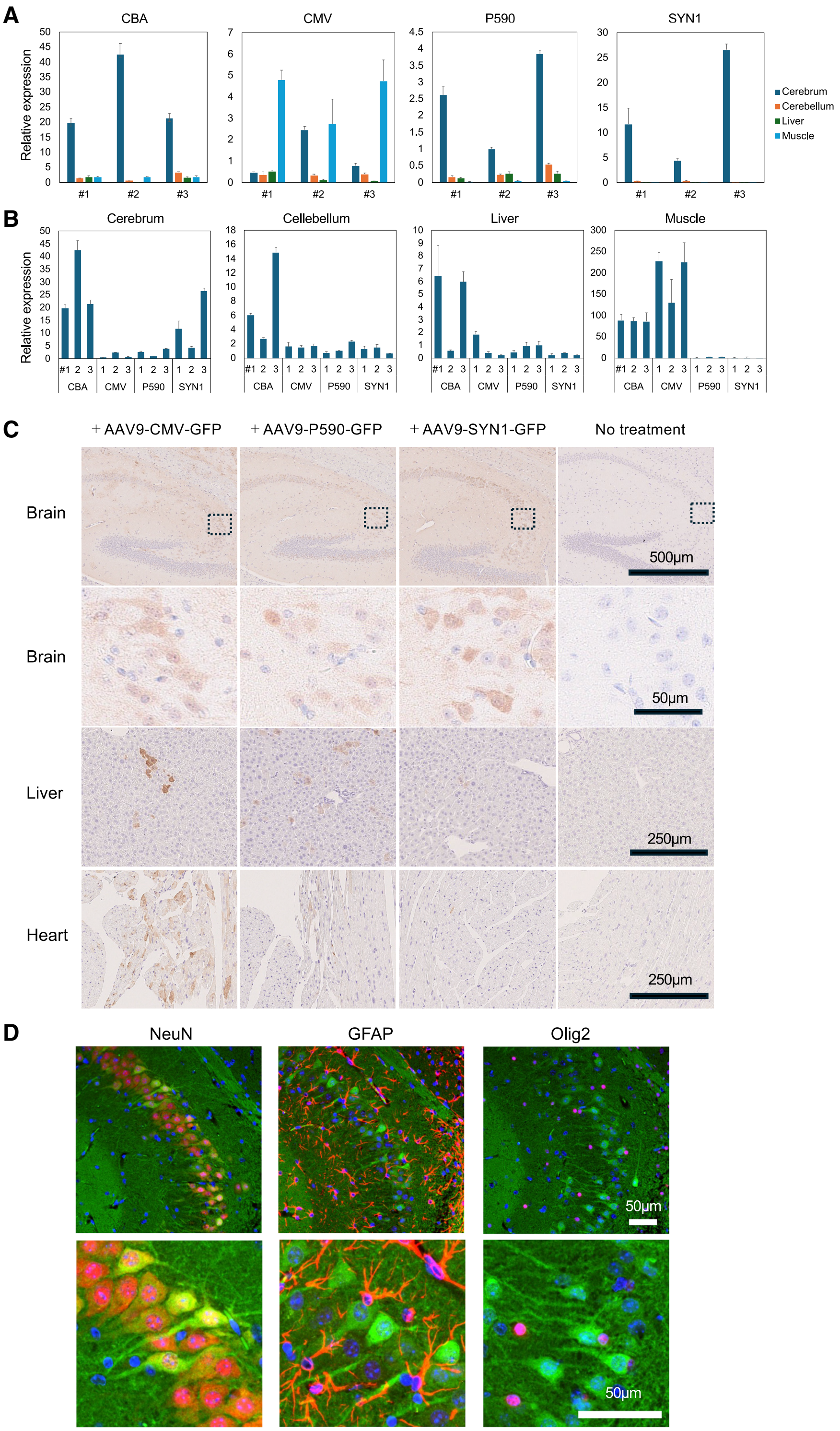

Figure 4. Comparison of PIGO tissue expression driven by various promoters using qPCR and tissue staining

(A and B) *hPIGO* expression was assessed by qPCR 1 year after ICV administration of AAV.GTX-*hPIGO* (1×10^{11} vg/mouse) with various promoters. (A) Relative expression of *hPIGO* in various tissues for each promotor. Data are presented as mean + SD; (B) Relative expression of *hPIGO* in each promotor across tissues. Data are presented as mean + SD. $n = 3$. (C) Comparison of GFP DAB staining after ICV injection of AAV9-*GFP* (1×10^{11} vg/mouse) driven by various promoters. These results are representative of

(legend continued on next page)

expression.[16] The Y446F and Y731F substitutions enhance transduction efficiency in the brain and retina, respectively.[16,23]

On the other hand, AAV9 is known to bind to cell-surface galactose,[24] and residues, such as D271, N272, Y446, N470, and W503, have been identified as key determinants of this interaction.[25] The AAV.GTX vector used in this study carries a mutation at Y446, which is one of the critical residues required for galactose binding; therefore, its substitution is expected to alter receptor interactions. Hoffmann et al. reported that mutations at Y446, N470, and W503 alter galactose binding and can shift tropism from the liver to other peripheral tissues, depending on the glycan profile of each tissue.[26] These findings suggest that the amino acid substitutions present in AAV.GTX likely modulate galactose-binding affinity and consequently influence tissue tropism. Among our treated mouse, only one case of hepatic cancer was observed in AAV.GTX-*CBA-hPIGO*-treated mice, whereas 3 of the 4 AAV9-*CBA-hPIGO*-treated mice developed hepatic cancer. This difference may be explained by the decreased affinity of AAV.GTX for galactose on the hepatocytes, although *PIGO* expression levels in the liver did not differ significantly between the two groups. Further studies will be required to determine the extent to which these alterations contribute to the vector's *in vivo* distribution and transduction profile.

It is known that AAV preferentially integrates into the Rian locus, leading to overexpression of proximal *microRNAs* and *Rtl1*, which can contribute to carcinogenesis.[15] This event is more frequently observed when using a high AAV dose, strong promoters such as the *CBA* promoter or the *TBG* promoter combined with the *CMV* enhancer, as well as neonatal AAV administration. Overexpression of *microRNAs* does not occur when other promoters are used, indicating that vector-encoded *cis*-regulatory sequences are responsible.[15] Although no human cases treated with an AAV vectors driven by *CBA* have developed liver cancer to date, a promoter that provides the minimum required expression of *PIGO* is considered safer.

Therefore, restoring PIGO expression to physiological levels across the body is desirable. The endogenous promoter *P590*, although driving lower expression than stronger promoters as shown by qPCR analysis, most effectively restored motor function in *PIGO* KI/KO mice. This suggests that PIGO, as an enzyme, can exert therapeutic effects even at relatively low expression levels. The functional recovery and improved survival observed in this study are remarkable, and these findings are expected to contribute to the development of a safe and promising therapeutic strategy for PIGO deficiency.

The expression pattern of GFP driven by each promoter displayed distinct characteristics. Because the vector was administered into the cerebral ventricles, all promoters yielded broad expression throughout the brain. However, the GFP driven by *CMV* promoter was strongly expressed in the heart and liver and showed a speckled staining pattern in the brain, which was clearly different from that produced by the other two promoters. As expected, *SYN1* promoter drove expression exclusively in neurons. Although the therapeutic effect of AAV-*SYN1-hPIGO* in model mice was slightly weaker than that achieved with the other systemically active promoters, it was generally effective. This suggests that most of the phenotypic abnormalities arise from impaired PIGO function in neurons. This interpretation is further supported by the fact that the endogenous promoter *P590*, which showed the strongest therapeutic effect, is expressed predominantly in neurons rather than glial cells.

Regarding the administration route, intrathecal delivery is considered safer than ICV administration in humans. Therefore, we evaluated IT-C administration in mice and compared its effectiveness with that of ICV delivery. In the model mice, the therapeutic effect of IT-C injection was comparable to that of ICV injection. However, the staining pattern of P590-GFP in the brain was different between the two routes: with IT-C administration, expression was observed in the olfactory bulb, cerebral cortex neurons, and cerebellar Purkinje cells, whereas ICV administration produced overall stronger expression, particularly in hippocampal neurons.

Considering these findings and the safety profile, IT-C administration appears to be the more appropriate route for human therapy. In both administration routes, lowering the dose reduced therapeutic efficacy, indicating that 1×10^{11} vg/mouse is the minimum required dose.

Thus, in the model mice, treatment at the neonatal stage (P1–P3) was highly effective, and most phenotypes were reversible. In humans, however, the diagnosis of IGD is typically delayed—ranging from several months of age to around 3 years—despite the availability of a screening method based on fluorescence-activated cell sorting (FACS) analysis of peripheral granulocytes, which is already established and covered by insurance in Japan (submitted). When the mice were treated at 4 weeks of age (corresponding to approximately 3–4 years of age in humans) with a 10-fold higher dose, therapeutic effects were partial but still significant, suggesting that this gene therapy could also be applicable for older children, potentially up to about 5 years of age. Treated mice exhibited approximately a 10-fold increase in body weight, suggesting that even at this higher dose the relative exposure per body weight would be comparable to that under lower dosing conditions. Moreover, AAV-associated liver tumors have been reported mainly after neonatal administration and are generally not observed in adult mice under standard conditions.[27] Therefore, liver tumor formation in our experimental setting appears unlikely, although longer-term observation would

analyses performed on three mice per group. The data including the other two mice are shown in the supplemental information (Figure S4). (D) Immunostaining analysis of the mouse hippocampus administered with AAV9-*P590-GFP* shown in (C). Merged images of GFP (green) and DAPI (blue). Neurons, astrocytes, and oligodendrocytes were visualized using Alexa Fluor 647-conjugated anti-NeuN, anti-GFAP, and anti-Olig2 antibodies, respectively (red) (C and D). AAV9 was used for this comparative study because vectors with the GTX capsid could not be outsourced due to licensing restrictions.

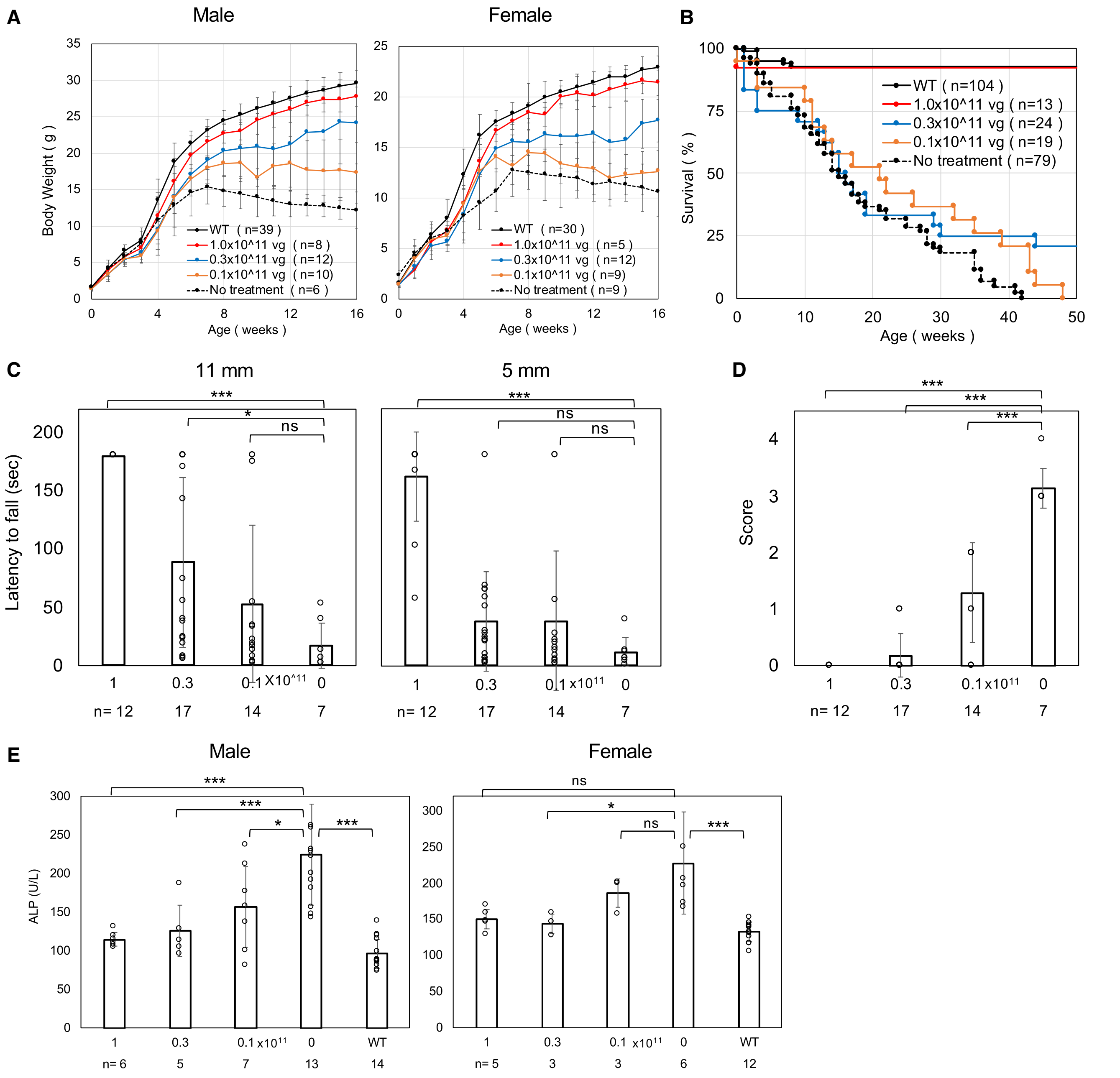

Figure 5. The effectiveness of *P590* promoter-driven AAV.GTX-*hPIGO* was dose dependent

(A) Comparison of weekly body weight in AAV-treated KI/KO mice and untreated KI/KO mice. Data are presented as mean ± SD. *n* indicates the number of animals. (B) Kaplan-Meier survival curves for each group. *n* indicates the number of animals. (C) Comparison of the latency to fall in the hanging wire test among groups. Data are presented as mean ± SD. *n* indicates the number of animals. Statistical analysis was performed using one-way ANOVA followed by Dunnett's post hoc test (*$p < 0.05$; ***$p < 0.001$; ns, not significant). For the 11 mm mesh (ANOVA $p = 5.8 \times 10^{-7}$), 1×10^{11} and 0.3×10^{11} vg/mouse differed from no treat ($p < 0.001$ and $p = 0.022$, respectively), whereas 0.1×10^{11} vg/mouse did not ($p = 0.37$). For the 5 mm mesh (ANOVA $p = 1.5 \times 10^{9}$), only 1×10^{11} vg/mouse differed from no treat ($p < 0.001$), whereas the other doses were not significant. (D) Comparison of tremor score among groups. Data are presented as mean ±SD. *n* indicates the number of animals. Statistical analysis was performed using one-way ANOVA followed by Dunnett's post hoc test (***$p < 0.001$). ANOVA $p = 5.6 \times 10^{-16}$; all treatment groups (1×10^{11}, 0.3×10^{11}, and 0.1×10^{11} vg/mouse) differed from no treat (all $p < 0.001$). (E) Amelioration of hyperphosphatasia. ALP activity was measured in plasma from mice older than 4 months. Data are presented as mean ± SD. *n* indicates the number of animals. Statistical analysis was performed using one-way ANOVA followed by Dunnett's post hoc test for males and Kruskal-Wallis test followed by Dunn's post hoc test for females (*$p < 0.05$; ***$p < 0.001$; ns, not significant). Males: ANOVA $p = 2.1 \times 10^{-7}$;1×10^{11} and 0.3×10^{11} vg/mouse and wild type differed from no treat ($p < 0.001$), whereas 0.1×10^{11} vg/mouse showed a modest effect ($p = 0.013$). Females: overall $p = 6.2 \times 10^{-4}$; wild type and 0.3×10^{11} vg/mouse differed from no treat $p < 0.001$, $p = 0.046$, respectively), whereas 1×10^{11} and 0.1×10^{11} vg/mouse did not ($p = 0.054$ and 0.70, respectively).

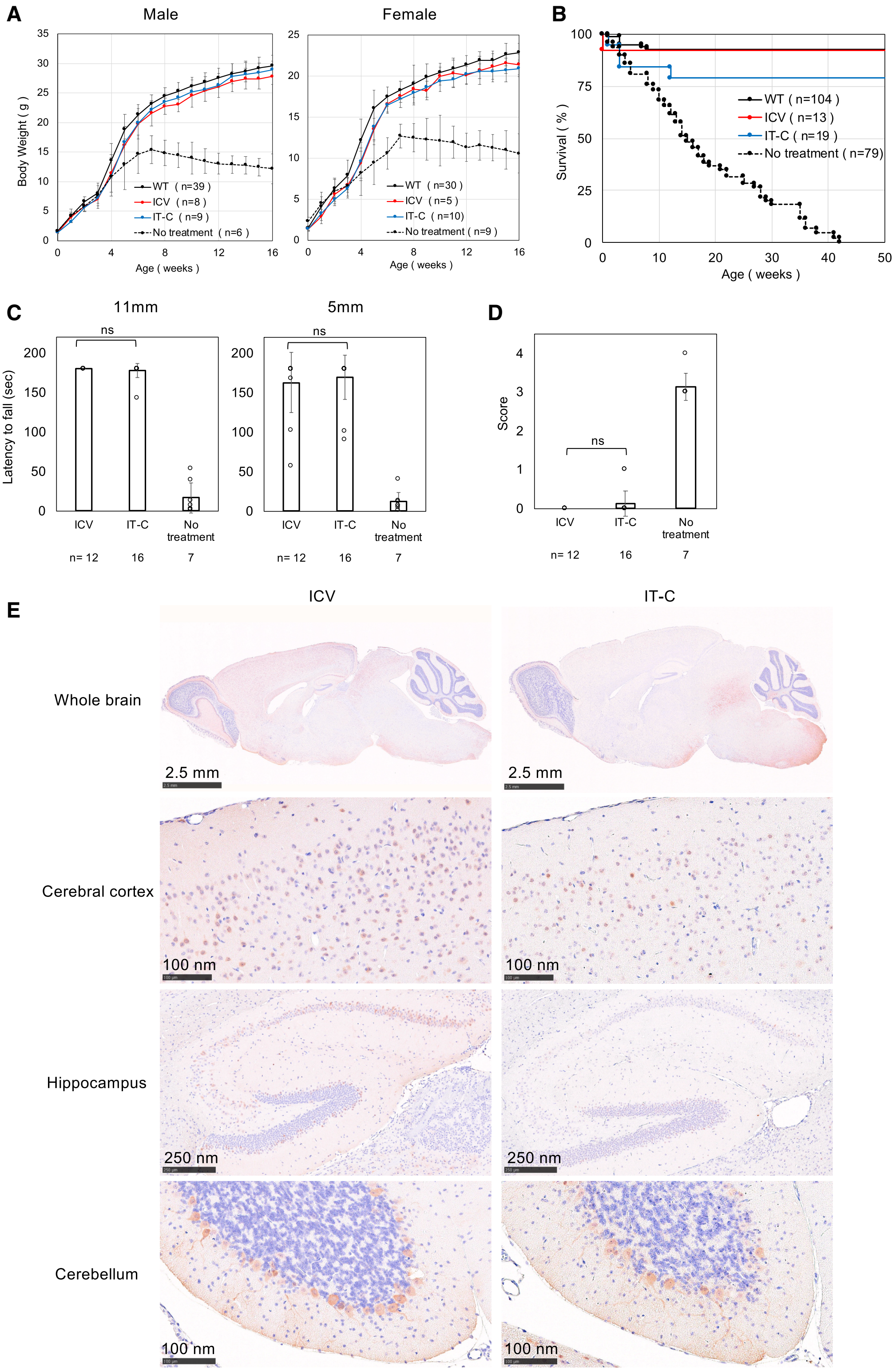

(legend on next page)

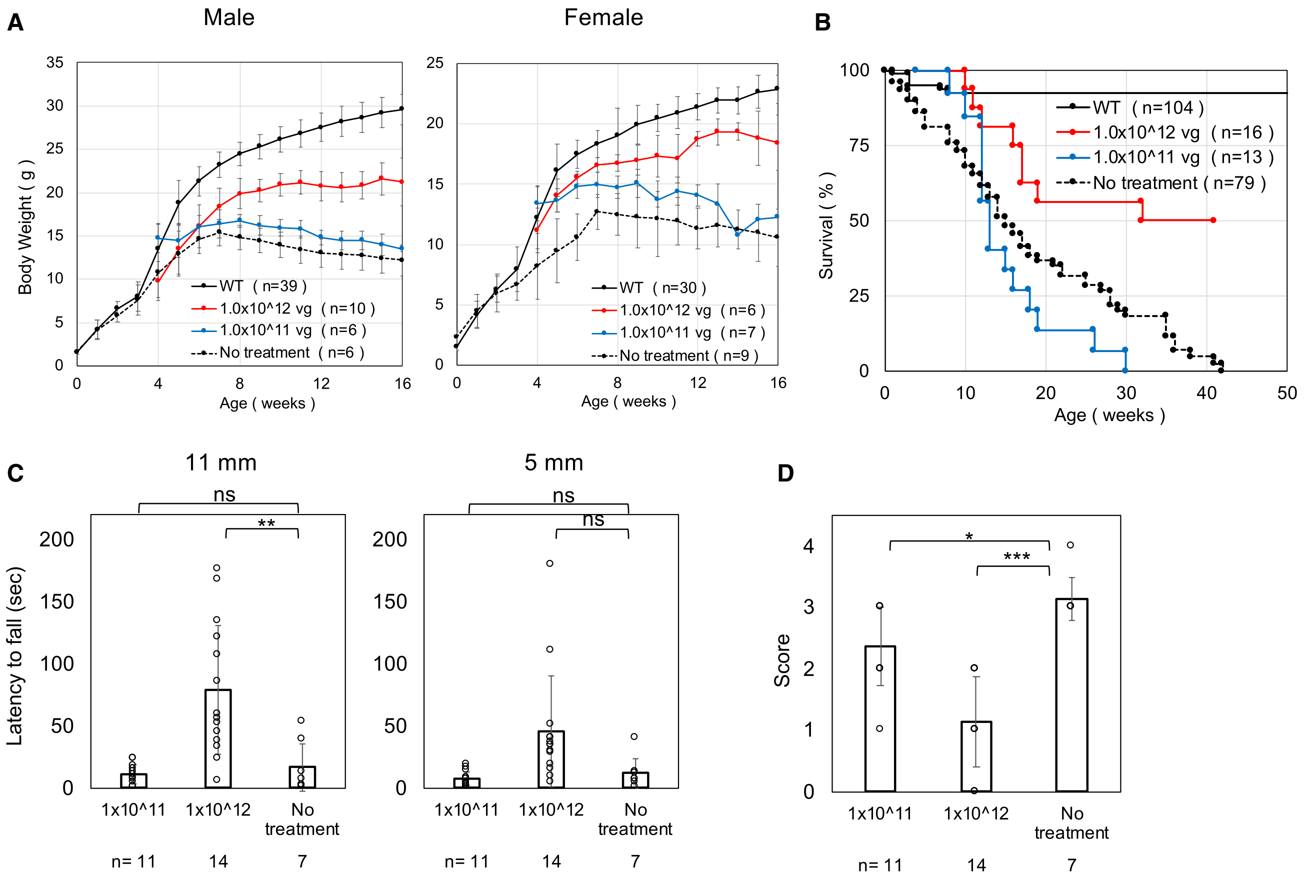

Figure 7. Administration of AAV9-*P590-hPIGO* at 4 weeks of age resulted in partial but significant symptom improvement

(A) Comparison of weekly body weight in mice treated at 4 weeks of age. Data are presented as mean ± SD. *n* indicates the number of animals. (B) Kaplan-Meier survival curves in mice treated at 4 weeks of age. *n* indicates the number of animals. (C) Comparison of the latency to fall in the hanging wire test in mice treated at 4 weeks of age. Data are presented as mean ± SD. *n* indicates the number of animals. Statistical analysis was performed using one-way ANOVA followed by Dunnett's post hoc test (**$p < 0.01$; ns, not significant). For the 11 mm mesh (ANOVA $p = 1.7 \times 10^{-4}$), 1×10^{12} vg/mouse differed from no treat ($p = 0.002$), whereas 1×10^{11} vg/mouse did not. For the 5 mm mesh (ANOVA $p = 1.5 \times 10^{-9}$), both doses did not. (D) Comparison of tremor score in mice treated at 4 weeks of age. Data are presented as mean ±SD. *n* indicates the number of animals. Statistical analysis was performed using one-way ANOVA followed by Dunnett's post hoc test (*$p < 0.05$; ***$p < 0.001$) ANOVA $p = 1 \times 10^{-6}$; both doses (1×10^{11} and 1×10^{12} vg/mouse) differ from no treat ($p = 0.04$ and $p < 0.001$, respectively). AAV9 was used for this study, which required a high titer for administration, because vectors with the GTX capsid could not be outsourced due to licensing restrictions.

be required to fully exclude this possibility. Nevertheless, early diagnosis and early treatment are desirable, and we are currently developing a diagnostic method suitable for inclusion in newborn screening programs.

In severe cases, patients present with multi-organ anomalies, such as diaphragmatic hernia, Hirschsprung disease, congenital heart defects, urogenital anomalies, and esophageal atresia.[28] These anomalies cannot be reproduced in mice and are unlikely to be treatable by the current gene-therapy approach.

Patients with IGD also frequently suffer from intractable seizures. Since administration of AAV-*PIGO* reduced seizure susceptibility to the convulsant in mice, it is possible that this therapy could help

Figure 6. IT-C administration of AAV.GTX-*hPIGO* was similarly effective to ICV administration

(A) Comparison of weekly body weight between ICV and IT-C administration. Data are presented as mean ± SD. *n* indicates the number of animals. (B) Kaplan-Meier survival curves of ICV and IT-C administration. *n* indicates the number of animals. (C) Comparison of the latency to fall in the hanging wire test between ICV and IT-C administration. Data are presented as mean ± SD. *n* indicates the number of animals. Statistical analysis was performed using Student's two-sided *t* test (ns, not significant). (D) Comparison of tremor score between ICV and IT-C administration. Data are presented as mean ± SD. *n* indicates the number of animals. Statistical analysis was performed using Student's two-sided *t* test (ns, not significant). (E) Comparison of DAB staining for GFP in tissue following ICV and IT-C administration of AAV.GTX-*P590-GFP* (0.3×10^{11} vg/mouse). The top photo shows the whole brain, the second the cerebral cortex, the third the hippocampus, and the bottom the cerebellum. These results are representative of analyses performed on three mice per group. The data including the other two mice are shown in the supplemental information (Figure S6).

control refractory seizures in patients, which would greatly improve their quality of life.

MATERIALS AND METHODS

Generation of AAV

Human *PIGO* cDNA was inserted into the pAAV-*CBA* plasmid to generate pAAV-*CBA-hPIGO*. The endogenous *P590*, *SYN1*, or *CMV* promoter was substituted for the *CBA* promoter to generate pAAV-*P590-hPIGO*, pAAV-*SYN1-hPIGO*, and pAAV-*CMV-hPIGO*, respectively. AAV particles were produced by co-transfecting pAAV-*hPIGO*, the AAV9 or AAV.GTX capsid plasmid, and the pAd5 helper plasmid (Addgene) into AAVpro 293T cells (Takara Bio, Kusatsu, Japan) using a lipofection-based method. Viral particles were purified by polyethylene glycol precipitation followed by iodixanol-gradient ultracentrifugation. Viral titers were determined by qPCR using TaqMan technology (Thermo Fisher Scientific). The production of AAV9-*P590-hPIGO*, which required a high titer for administration to 4-week-old mice, was outsourced to VectorBuilder, together with the production of vectors for AAV9-*P590-GFP*, -*CMV-GFP*, and -*SYN1-GFP*. Vectors using the GTX capsid could not be outsourced due to licensing restrictions.

Animals

All mice were C57BL/6 background and maintained in SPF under a 12 h light/12 h dark cycle with food and water provided ad libitum. Temperature and humidity were within the recommended range (20°C–24°C and 40%–60%, respectively). All animal procedures were approved by the Animal Care and Use Committee of the Research Institute for Microbial Diseases, The University of Osaka, Japan; all methods were carried out in accordance with the approved guidelines. Pigo heterozygous KI mice and KO mice were maintained by mating with C57BL/6 mice.

The model mouse of PIGO deficiency and its genotyping

The PIGO-deficient model mouse was established previously.[10] For the gene therapy experiments, compound heterozygous mice were generated by crossing heterozygous KI mice carrying the patient's mutation Thr130Asn with heterozygous KO mice. Genotyping was performed as described previously; briefly, genomic DNA was extracted from mouse tail biopsies and amplified by polymerase chain reaction (PCR) using PrimeSTAR GXL Premix Fast DNA polymerase (Takara Bio) with the following primers.

Primer 1: 5′TTGCCACCCTGGAAATGTTG3′

Primer 2: 5′TAGAGGTGTTCCAAGATGCCG3′

Primer 3: 5′CACATTTCCCCGAAAAGTGCCAC3′

PCR products were digested with SalI-HF (New England Biolabs) and separated on a 1% agarose gel. KI alleles were identified by the presence of digested bands at 1,021 bp and 761 bp, whereas KO alleles were identified by the bands at 878 bp and 761 bp.

ICV AAV injection

The newborn (P1–P3) KI/KO mice were subjected to ICV injection of AAV. Pups were anesthetized by placing them on ice for 1 min and injected using a 33G needle attached to a 25-μL syringe (Hamilton, #65460–10). The injection site was located 1 mm lateral to the midline, 0.3 mm posterior to the bregma, and 1.5 mm in depth. Injected volume was around 5 μL. After injection pups were allowed to rewarm and recover for 2–3 min before being returned to their cage.

For ICV injections in 4-week-old mice, animals were anesthetized with isoflurane and the scalp was incised to expose the skull. AAV was administered using the same 33G needle and 25-μL (Hamilton, #65460–10) at the same coordinates: 1 mm lateral to the midline, 0.3 mm posterior to the bregma, and 1.5 mm in depth. The incision was then closed and the mice were allowed to recover.

IT-C AAV injection

Newborn (P1–P3) KIKO mice were subjected to IT-C AAV injection. Pups were anesthetized by placing them on ice for 1 min and injected using a 33G needle attached to 25-μL syringe (Hamilton, #65460–10). With the pup's head maximally flexed, the needle was inserted into the center of the neck depression at a 45° angle to a depth of 1.5 mm. After injection, pups were allowed to rewarm and recover for 2–3 min before being returned to their cage.

Animal behavioral analysis

Muscle weakness and coordination deficit were assessed using the four-limb hanging test (5 mm and 11 mm mesh) at 8 weeks of age. The latency to fall was recorded with a 3-min cut-off time. The performance of AAV-treated mice was compared with that of wild-type controls and untreated controls.

Tremor data were quantitatively scored according to the following criteria.[29]

0 points: no tremor.

1 point: occasional tremors affecting only the head and neck.

2 points: intermittent tremors affecting the entire body.

3 points: persistent tremors affecting the entire body and tail.

4 points: severe, persistent tremors rendering the animal unable to stand and/or walk.

Tremor assessment was performed by two observers, one of whom was blinded.

Measuring ALP activity

Whole blood was collected in a heparin-treated tube from the mouse tail artery, and plasma was separated by centrifugation. Plasma ALP activity was measured in the clinical laboratory at BIKEN using the

JSCC-recommended method with 4-nitrophenylphosphate (4-NPP) as the substrate. As the measurement method was changed to the IFCC-recommended method during the course of the study, values obtained by the JSCC-recommended method were converted using an appropriate conversion formula. As ALP activity is physiologically high in young mice, only animals older than 4 months were analyzed (Figure S7).

Measurement of virus-derived PIGO expression in various tissues and endogenous Rtl1 in liver cancers

At 1 year of age, AAV-treated mice (n = 3) were euthanized, and tissues were collected. Total RNA was isolated with an RNeasy kit (Qiagen) after tissue homogenization. RNA was reverse transcribed using a Superscript VILO kit (Thermo Fisher Scientific). qPCR was performed using SYBR Green PCR mater mix (Thermo Fisher Scientific) and gene-specific primers on the Step One Plus Real-Time PCR System (Thermo Fisher Scientific) with the comparative Ct method. The primers used were as follows.

- *hPIGO*: 5′-GCCACGCCATAGTGGAAGACA-3′ and 5′-CACCAGGGAAAAGGTCTTTCCAGG-3′
- *mRtl1*: 5′-TACTGCTCTTGGTGAGAGTGGACCC-3′ and 5′-GGAGCCACTTCATGCCTAAGACGA-3′
- Endogenous control (*mTbp*, TATA-box-binding protein): 5′-TATGACCCCTATCACTCCTG-3′ and 5′-TTCTTCACTCTTGGCTCCTGT-3′

Copy numbers of *hPIGO mRNA* and *mRtl1 mRNA* were normalized to *mTbp* expression.

Analysis of promotor activity *in vitro*

The activities of *PIGO* endogenous promoters of various lengths, along with several other candidate promoters were evaluated in HEK293 cells and Neuro2a cells using Dual-Luciferase Reporter Assay System (Promega). Candidate promoters were inserted into pAAV-*hPIGO* construct and were transfected into *Pigo*-KO Neuro2a cells to assess rescue of cell-surface expression of the GPI-anchored protein CD24, which was quantified by flow cytometry (FACS).

Evaluation of PTZ-induced seizures

Mice were injected intraperitoneally with the dose of 20 mg/kg PTZ (Cayman). The behaviors were observed for 30 min after administration and classified and scored using modified Racine scale.[30]

The score according to the following criteria[31]:

0 points: no abnormality (normal).

1 point: exploring, sniffing, and grooming ceased, becoming motionless.

2 points: head nodding, facial and forelimb clonus (short myoclonic jerk).

3 points: “continuous myoclonic jerk (clonic convulsion),” myoclonic jerks of the head and neck, with brief twitching movement, or repetitive movements with head bobbing or “wet-dog shakes,” tail rigidity.

4 points: forelimb or forelimb and hindlimb clonus, reciprocal forepaw padding, hindlimb abduction, continuous rearing and falling, Straub tail response (specially for mouse), kangaroo posture.

5 points: tonic convulsion.

Seizures assessment was performed by two observers, one of whom was blinded.

Histological analysis of the mouse tissues

Mice at 4 months of age were deeply anesthetized and transcardially perfused with 20–30 mL of PBS/1% heparin followed by 50 mL of 4% paraformaldehyde (PFA)/PBS. The brain, liver, and heart were taken and fixed in 4% PFA/PBS in 4°C for 48 h. Tissues were then washed twice with PBS at room temperature (RT), followed by three washes with 70% ethanol at RT. Paraffin embedding, sectioning and staining were outsourced to GenoStaff Co.,Ltd.

For DAB staining, the primary antibody used was goat polyclonal anti-GFP (0.1 μg/mL Abcam, #ab6673), with goat IgG (R&D, #AB-108-C) as a negative control.

For fluorescent double staining, the following primary antibodies were used.

- Goat polyclonal anti-GFP (0.5 μg/mL Abcam, #ab6673) and goat IgG (R&D, #AB-108-C) as a negative control
- Rabbit monoclonal anti-NeuN (D4G4O) (0.05 μg/mL CST, #24307) and rabbit IgG (Vector, #I-1000) as a negative control
- Rat monoclonal anti-GFAP (2.2B10) (0.4 μg/mL Invitrogen, #13–0300) and rat IgG2a (R&D, #MAB006) as a negative control
- Rabbit monoclonal anti-Olig2 (E6G6Q) (0.08 μg/mL CST, #65915) and rabbit IgG (Vector, #I-1000) as a negative control.

The second antibodies used were goat anti-goat IgG Alexa 488 (Invitrogen, #A11055), anti-rabbit IgG Alexa 647 (Invitrogen, #A31573), and anti-rat IgG Alexa 647 (Invitrogen, #A21472).

Statistical analysis

Statistical analyses were performed using one-way ANOVA followed by Dunnett’s post hoc test. For the data with a small sample size (n = 3), the Kruskal-Wallis test followed by Dunn’s post hoc test was used.

Between two groups with sufficient sample size, the Student’s two-sided t test was used.

DATA AND CODE AVAILABILITY

All of the data are available from the corresponding author on reasonable request.

ACKNOWLEDGMENTS

This work was supported by a grant from the Ministry and Health, Labour, and Welfare, the Practical Research Project for Rare/Intractable Diseases from the Japan Agency for Medical Research and Development (AMED) and JSPS KAKENHI (23FC1033, JP22ek0109614, JP25ek0109808, JP23bm1223019, and 23K05695 to Y.M.).

AUTHOR CONTRIBUTIONS

Y.M., S.U., S.M., K.C.M., and T.K. designed the study. Y.M., K.I., M.I., N.T., and S.U. acquired the data and conducted the experiments. K.I., S.M., and S.L. made the AAV. Y.M., T.K., S.L., K.C.M., and S.U. wrote the paper.

DECLARATION OF INTERESTS

Y.M. and S.M. are inventors on patents for AAV vectors used in this paper. S.M. owns equity in a gene therapy company (ONODERA GT Pharma, Co., Ltd.) that commercializes the use of AAV vectors for gene therapy applications. K.C.M. recently founded Neela Therapeutics, an independent clinical-stage biotech company focused on pediatric CNS disorders.

DECLARATION OF GENERATIVE AI AND AI-ASSISTED TECHNOLOGIES IN THE WRITING PROCESS

During the preparation of this work, the authors used ChatGPT for English proofreading. After using this tool/service, we reviewed and edited the content as needed and take full responsibility for the content of the publication.

SUPPLEMENTAL INFORMATION

Supplemental information can be found online at https://doi.org/10.1016/j.omta.2026.201724.

REFERENCES

1. Kinoshita, T. (2020). Biosynthesis and biology of mammalian GPI-anchored proteins. Open Biol. *10*, 190290. https://doi.org/10.1098/rsob.190290.

2. Almeida, A.M., Murakami, Y., Layton, D.M., Hillmen, P., Sellick, G.S., Maeda, Y., Richards, S., Patterson, S., Kotsianidis, I., Mollica, L., et al. (2006). Hypomorphic promoter mutation in PIGM causes inherited glycosylphosphatidylinositol deficiency. Nat. Med. *12*, 846–851.

3. Almeida, A.M., Murakami, Y., Baker, A., Maeda, Y., Roberts, I.A.G., Kinoshita, T., Layton, D.M., and Karadimitris, A. (2007). Targeted therapy for inherited GPI deficiency. N. Engl. J. Med. *356*, 1641–1647.

4. Murakami, Y. (2025). Biosynthesis of GPI anchored proteins, its deficiencies and treatment. J. Hum. Genet. https://doi.org/10.1038/s10038-025-01379-1.

5. Tanigawa, J., Nabatame, S., Tominaga, K., Nishimura, Y., Maegaki, Y., Kinosita, T., Murakami, Y., and Ozono, K. (2021). High-dose pyridoxine treatment for inherited glycosylphosphatidylinositol deficiency. Brain Dev. *43*, 680–687. https://doi.org/10.1016/j.braindev.2021.02.007.

6. Waymire, K.G., Mahuren, J.D., Jaje, J.M., Guilarte, T.R., Coburn, S.P., and MacGregor, G.R. (1995). Mice lacking tissue non-specific alkaline phosphatase die from seizures due to defective metabolism of vitamin B-6. Nat. Genet. *11*, 45–51.

7. Kuki, I., Takahashi, Y., Okazaki, S., Kawawaki, H., Ehara, E., Inoue, N., Kinoshita, T., and Murakami, Y. (2013). Case report on vitamin B6 responsive epilepsy due to inherited GPI deficiency. Neurology *81*, 1467–1469. https://doi.org/10.1212/WNL.0b013e3182a8411a.

8. Hyland, K., Shoffner, J., and Heales, S.J. (2010). Cerebral folate deficiency. J. Inherit. Metab. Dis. *33*, 563–570. https://doi.org/10.1007/s10545-010-9159-6.

9. Messina, M., Manea, E., Cullup, T., Tuschl, K., and Batzios, S. (2023). Hyperphosphatasia with mental retardation syndrome 3: Cerebrospinal fluid abnormalities and correction with pyridoxine and Folinic acid. JIMD Rep. *64*, 42–52. https://doi.org/10.1002/jmd2.12347.

10. Kuwayama, R., Suzuki, K., Nakamura, J., Aizawa, E., Yoshioka, Y., Ikawa, M., Nabatame, S., Inoue, K.I., Shimmyo, Y., Ozono, K., et al. (2022). Establishment of mouse model of inherited PIGO deficiency and therapeutic potential of AAV-based gene therapy. Nat. Commun. *13*, 3107. https://doi.org/10.1038/s41467-022-30847-x.

11. Hong, Y., Maeda, Y., Watanabe, R., Inoue, N., Ohishi, K., and Kinoshita, T. (2000). Requirement of PIG-F and PIG-O for transferring phosphoethanolamine to the third mannose in glycosylphosphatidylinositol. J. Biol. Chem. *275*, 20911–20919.

12. Murakami, Y., Umeshita, S., Imanishi, K., Yoshioka, Y., Ninomiya, A., Sunabori, T., Likhite, S., Koike, M., Meyer, K.C., and Kinoshita, T. (2024). AAV-based gene therapy ameliorated CNS-specific GPI defect in mouse models. Mol. Ther. Methods Clin. Dev. *32*, 101176. https://doi.org/10.1016/j.omtm.2023.101176.

13. Chan, K.Y., Jang, M.J., Yoo, B.B., Greenbaum, A., Ravi, N., Wu, W.L., Sánchez-Guardado, L., Lois, C., Mazmanian, S.K., Deverman, B.E., and Gradinaru, V. (2017). Engineered AAVs for efficient noninvasive gene delivery to the central and peripheral nervous systems. Nat. Neurosci. *20*, 1172–1179. https://doi.org/10.1038/nn.4593.

14. Huang, Q., Chan, K.Y., Tobey, I.G., Chan, Y.A., Poterba, T., Boutros, C.L., Balazs, A.B., Daneman, R., Bloom, J.M., Seed, C., and Deverman, B.E. (2019). Delivering genes across the blood-brain barrier: LY6A, a novel cellular receptor for AAV-PHP.B capsids. PLoS One *14*, e0225206. https://doi.org/10.1371/journal.pone.0225206.

15. Chandler, R.J., LaFave, M.C., Varshney, G.K., Trivedi, N.S., Carrillo-Carrasco, N., Senac, J.S., Wu, W., Hoffmann, V., Elkahloun, A.G., Burgess, S.M., and Venditti, C.P. (2015). Vector design influences hepatic genotoxicity after adeno-associated virus gene therapy. J. Clin. Investig. *125*, 870–880. https://doi.org/10.1172/JCI79213.

16. Iida, A., Takino, N., Miyauchi, H., Shimazaki, K., and Muramatsu, S.i. (2013). Systemic delivery of tyrosine-mutant AAV vectors results in robust transduction of neurons in adult mice. BioMed Res. Int. *2013*, 974819. https://doi.org/10.1155/2013/974819.

17. (2025). Phase I/II Study of Gene Therapy for GLUT1 deficiency. https://jrct.mhlw.go.jp/en-latest-detail/jRCT2033240749.

18. Nozaki, M., Ohishi, K., Yamada, N., Kinoshita, T., Nagy, A., and Takeda, J. (1999). Developmental abnormalities of glycosylphosphatidylinositol-anchor-deficient embryos revealed by Cre/loxP system. Lab. Invest. *79*, 293–299.

19. Foust, K.D., Nurre, E., Montgomery, C.L., Hernandez, A., Chan, C.M., and Kaspar, B.K. (2009). Intravascular AAV9 preferentially targets neonatal neurons and adult astrocytes. Nat. Biotechnol. *27*, 59–65. https://doi.org/10.1038/nbt.1515.

20. Giannelli, S.G., Luoni, M., Iannielli, A., Middeldorp, J., Philippens, I., Bido, S., Körbelin, J., and Broccoli, V. (2024). New AAV9 engineered variants with enhanced neurotropism and reduced liver off-targeting in mice and marmosets. iScience *27*, 109777. https://doi.org/10.1016/j.isci.2024.109777.

21. Moyer, T.C., Hoffman, B.A., Chen, W., Shah, I., Ren, X.Q., Knox, T., Liu, J., Wang, W., Li, J., Khalid, H., et al. (2025). Highly conserved brain vascular receptor ALPL mediates transport of engineered AAV vectors across the blood-brain barrier. Mol. Ther. *33*, 3902–3916. https://doi.org/10.1016/j.ymthe.2025.04.046.

22. Horowitz, E.D., Finn, M.G., and Asokan, A. (2012). Tyrosine cross-linking reveals interfacial dynamics in adeno-associated viral capsids during infection. ACS Chem. Biol. *7*, 1059–1066. https://doi.org/10.1021/cb3000265.

23. Petrs-Silva, H., Dinculescu, A., Li, Q., Min, S.H., Chiodo, V., Pang, J.J., Zhong, L., Zolotukhin, S., Srivastava, A., Lewin, A.S., and Hauswirth, W.W. (2009). High-efficiency transduction of the mouse retina by tyrosine-mutant AAV serotype vectors. Mol. Ther. *17*, 463–471. https://doi.org/10.1038/mt.2008.269.

24. Shen, S., Bryant, K.D., Brown, S.M., Randell, S.H., and Asokan, A. (2011). Terminal N-linked galactose is the primary receptor for adeno-associated virus 9. J. Biol. Chem. *286*, 13532–13540. https://doi.org/10.1074/jbc.M110.210922.

25. Bell, C.L., Gurda, B.L., Van Vliet, K., Agbandje-McKenna, M., and Wilson, J.M. (2012). Identification of the galactose binding domain of the adeno-associated virus serotype 9 capsid. J. Virol. *86*, 7326–7333. https://doi.org/10.1128/JVI.00448-12.

26. Hoffman, J.A., Denton, N., Sims, J.J., Meggersee, R., Zhang, Z., Olagbegi, K., and Wilson, J.M. (2024). Modulation of AAV9 Galactose Binding Yields Novel Gene Therapy Vectors and Predicts Cross-Species Differences in Glycan Avidity. Hum. Gene Ther. *35*, 734–753. https://doi.org/10.1089/hum.2024.050.

27. Dalwadi, D.A., Torrens, L., Abril-Fornaguera, J., Pinyol, R., Willoughby, C., Posey, J., Llovet, J.M., Lanciault, C., Russell, D.W., Grompe, M., and Naugler, W.E. (2021). Liver Injury Increases the Incidence of HCC following AAV Gene Therapy in Mice. Mol. Ther. *29*, 680–690. https://doi.org/10.1016/j.ymthe.2020.10.018.

28. Tanigawa, J., Mimatsu, H., Mizuno, S., Okamoto, N., Fukushi, D., Tominaga, K., Kidokoro, H., Muramatsu, Y., Nishi, E., Nakamura, S., et al. (2017). Phenotype-genotype correlations of PIGO deficiency with variable phenotypes from infantile lethality to mild learning difficulties. Hum. Mutat. *38*, 805–815. https://doi.org/10.1002/humu.23219.

29. Zhang, R.X., Xu, J.T., Zhong, H.J., Cai, Y.L., Zhuang, Y.P., Xie, Y.T., and He, X.X. (2023). Gut microbiota from essential tremor patients aggravates tremors in mice. Front. Microbiol. *14*, 1252795. https://doi.org/10.3389/fmicb.2023.1252795.

30. Van Erum, J., Van Dam, D., and De Deyn, P.P. (2019). PTZ-induced seizures in mice require a revised Racine scale. Epilepsy Behav. *95*, 51–55. https://doi.org/10.1016/j.yebeh.2019.02.029.

31. Itoh, K., Watanabe, M., Yoshikawa, K., Kanaho, Y., Berliner, L.J., and Fujii, H. (2004). Magnetic resonance and biochemical studies during pentylenetetrazole-kindling development: the relationship between nitric oxide, neuronal nitric oxide synthase and seizures. Neuroscience *129*, 757–766. https://doi.org/10.1016/j.neuroscience.2004.09.025.

OMTA, Volume *34*

Supplemental information

Optimized AAV vector enables potent therapeutic rescue of inherited glycosylphosphatidylinositol deficiency in mice

Saori Umeshita, Kae Imanishi, Shibi Likhite, Mika Ito, Naomi Takino, Kathrin C. Meyer, Taroh Kinoshita, Shinichi Muramatsu, and Yoshiko Murakami

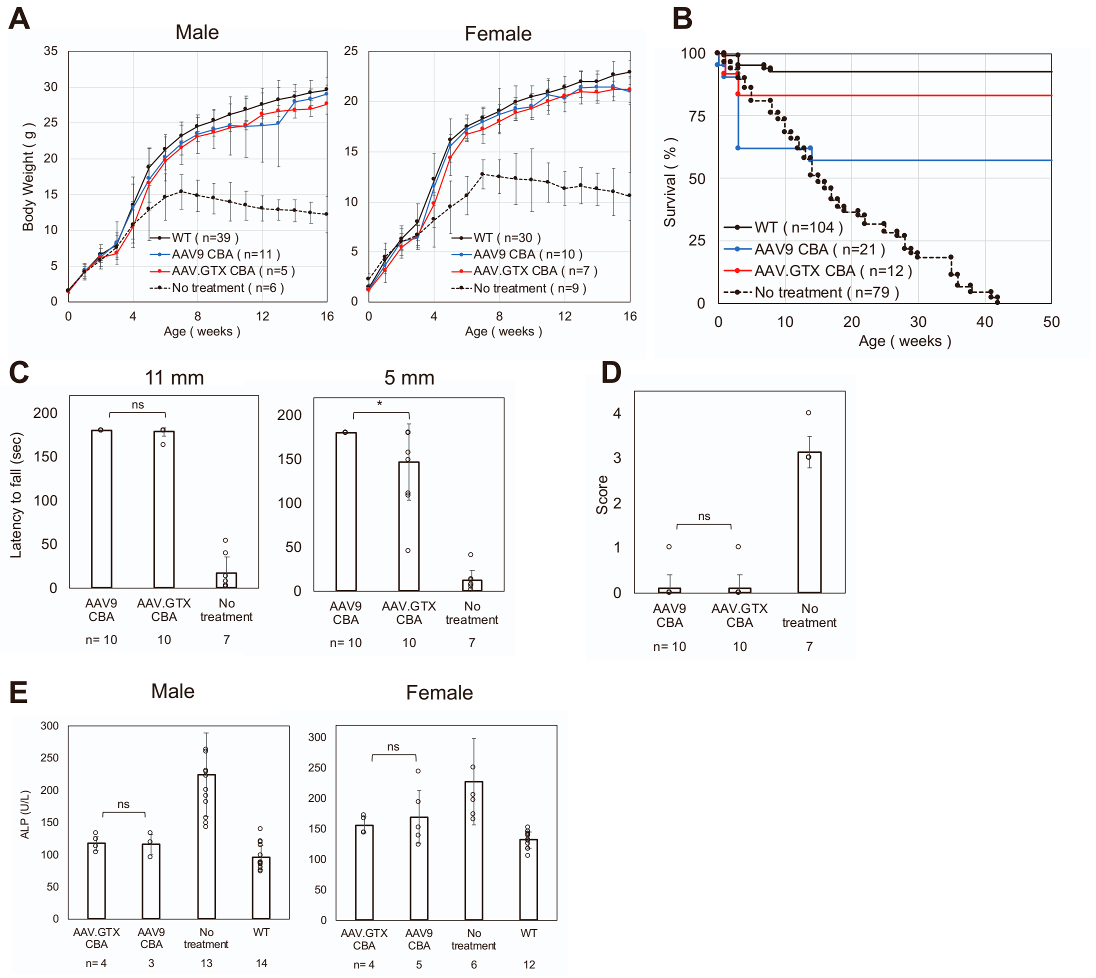

Figure S1. Comparison of AAV9 and AAV.GTX

Intracerebroventricular administration was performed once at 1×10^{11} vg/mouse on postnatal days 1–3.

A. Comparison of weekly body weight between AAV9-*CBA-hPIGO* treated KI/KO mice and AAV.GTX-*CBA-hPIGO* treated KI/KO mice. Data are presented as mean ± SD. *n* indicates the number of animals.

B. Kaplan-Meier survival curves for each group. *n* indicates the number of animals.

C. Comparison of latency to fall in the hanging wire test among groups. Data are presented as mean ± SD. *n* indicates the number of animals. (ns: not significant; **p*<0.05, Student's two-sided *t*-test) *P*-value for 5mm mesh: AAV9 vs. AAV.GTX, 3.4×10^{-2}.

D. Comparison of tremor score among groups. Data are presented as mean ± SD. *n* indicates the number of animals. (ns: not significant, two-sided *t*-test).

E. Amelioration of hyperphosphatasia. ALP activity was measured in plasma from mice older than 4 months. Data are presented as mean ± SD. *n* indicates the number of animals. (ns: not significant, Student's two-sided *t*-test)

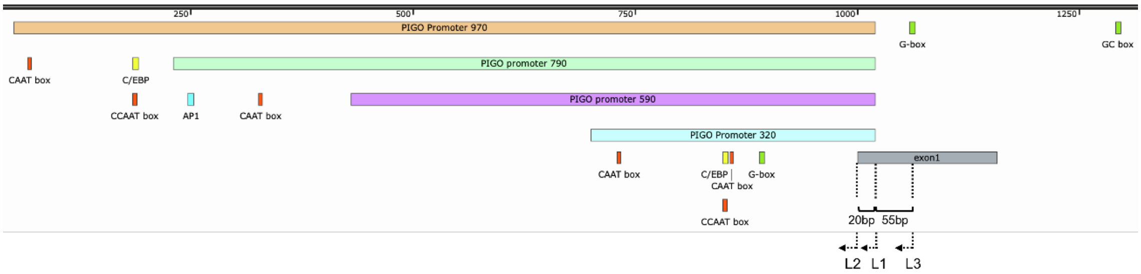

Figure S2. Design of Human Endogenous Promoter of *PIGO* Gene

The transcription factor binding sites were predicted by JASPR, an open-access database. (ref *Nucleic Acids Research*, gkaf1209, https://doi.org/10.1093/nar/gkaf1209)

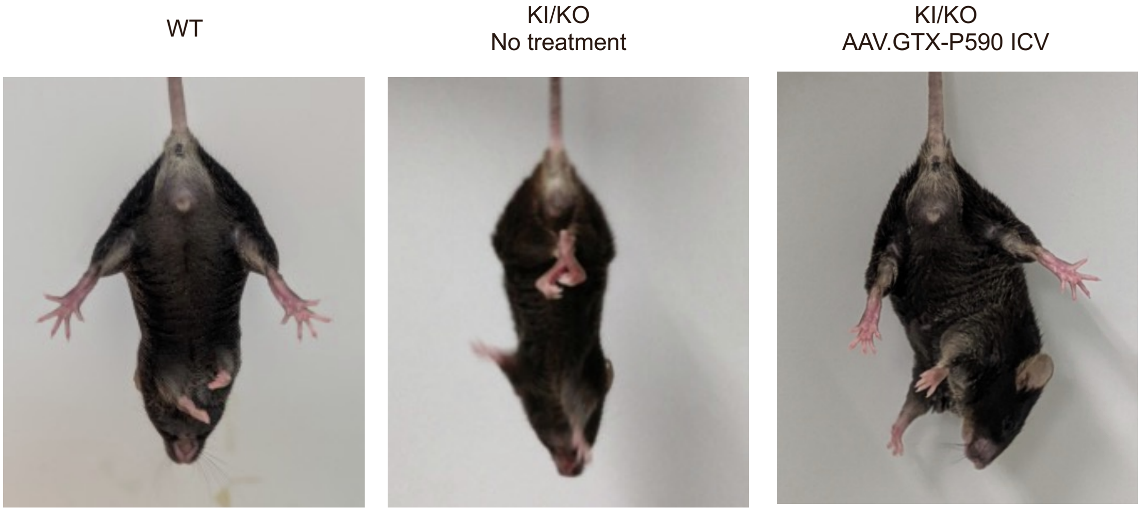

Figure S3. Hindlimb clasping in AAV-treated and untreated KI/KO mice

When mice were suspended by their tail, untreated mice showed hindlimb clasping, while AAV.GTX-*P590* treated mice appeared normal.

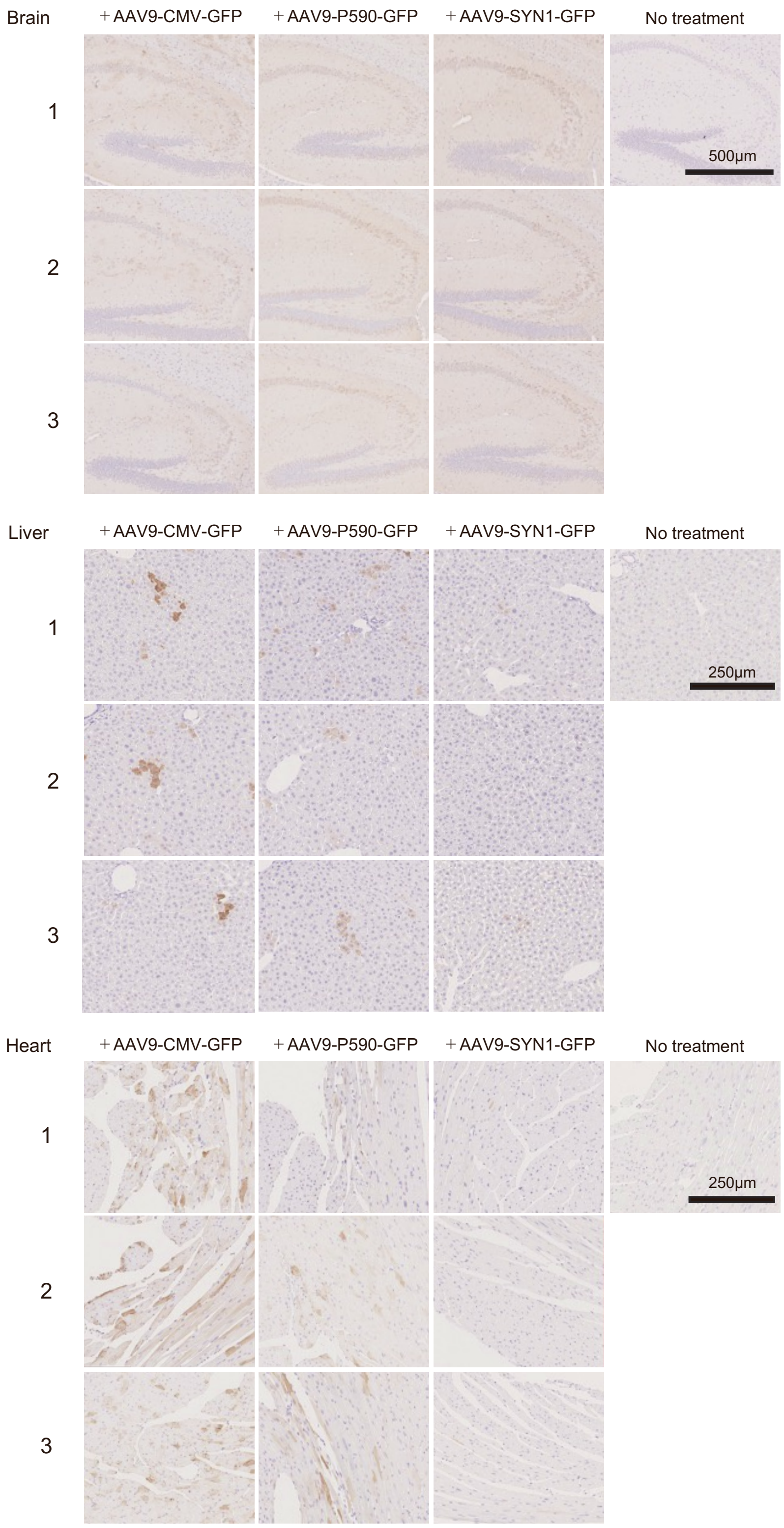

Figure S4. Comparison of GFP DAB staining after ICV injection of AAV9-*GFP* (1 × 10^{11} vg/mouse) driven by various promoters

The results show three cases in each group and one case in the negative control.

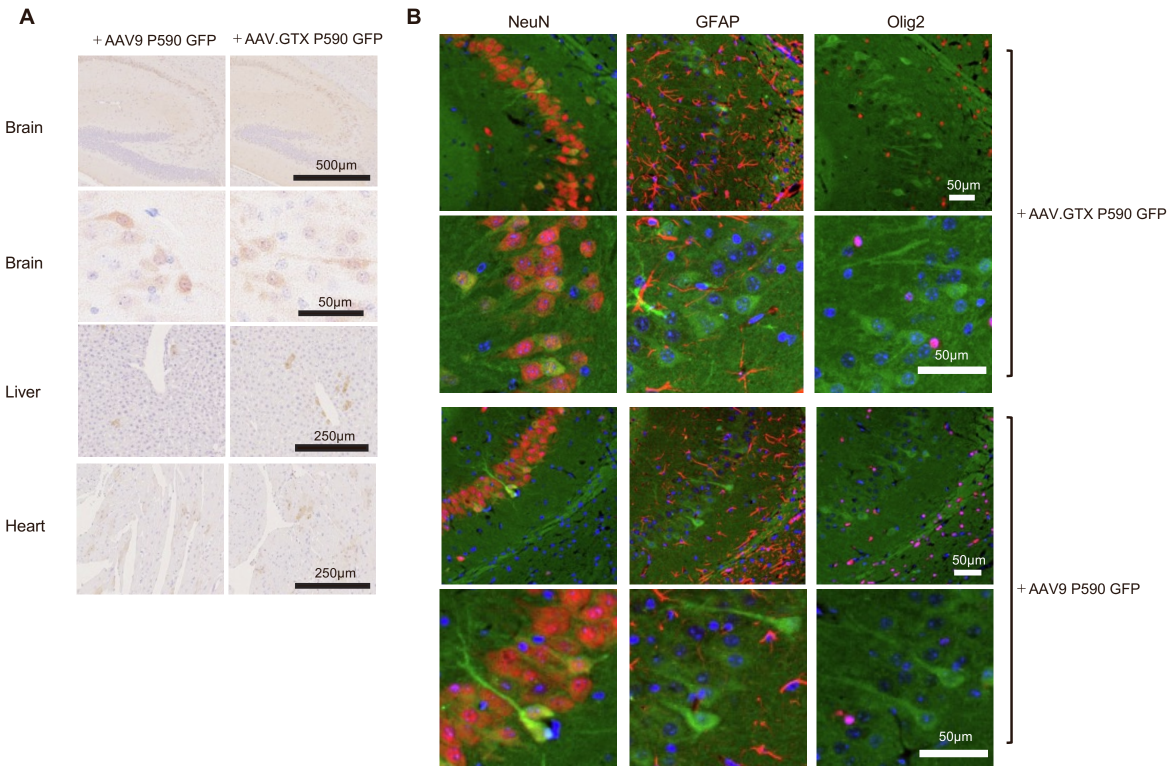

Figure S5. Comparison of GFP tissue staining after ICV injection of AAV.GTX-*P590-GFP* and AAV9-*P590-GFP* (0.3 × 10^{11} vg/mouse)

A. DAB staining for GFP expression.

B. Immunostaining analysis of the mouse hippocampus shown in A. The panels show merged images of GFP (green) and DAPI (blue). Neurons, astrocytes and oligodendrocytes were visualized using Alexa Fluor™ 647-conjugated anti-NeuN, anti-GFAP and anti-Olig2 antibodies, respectively (red).

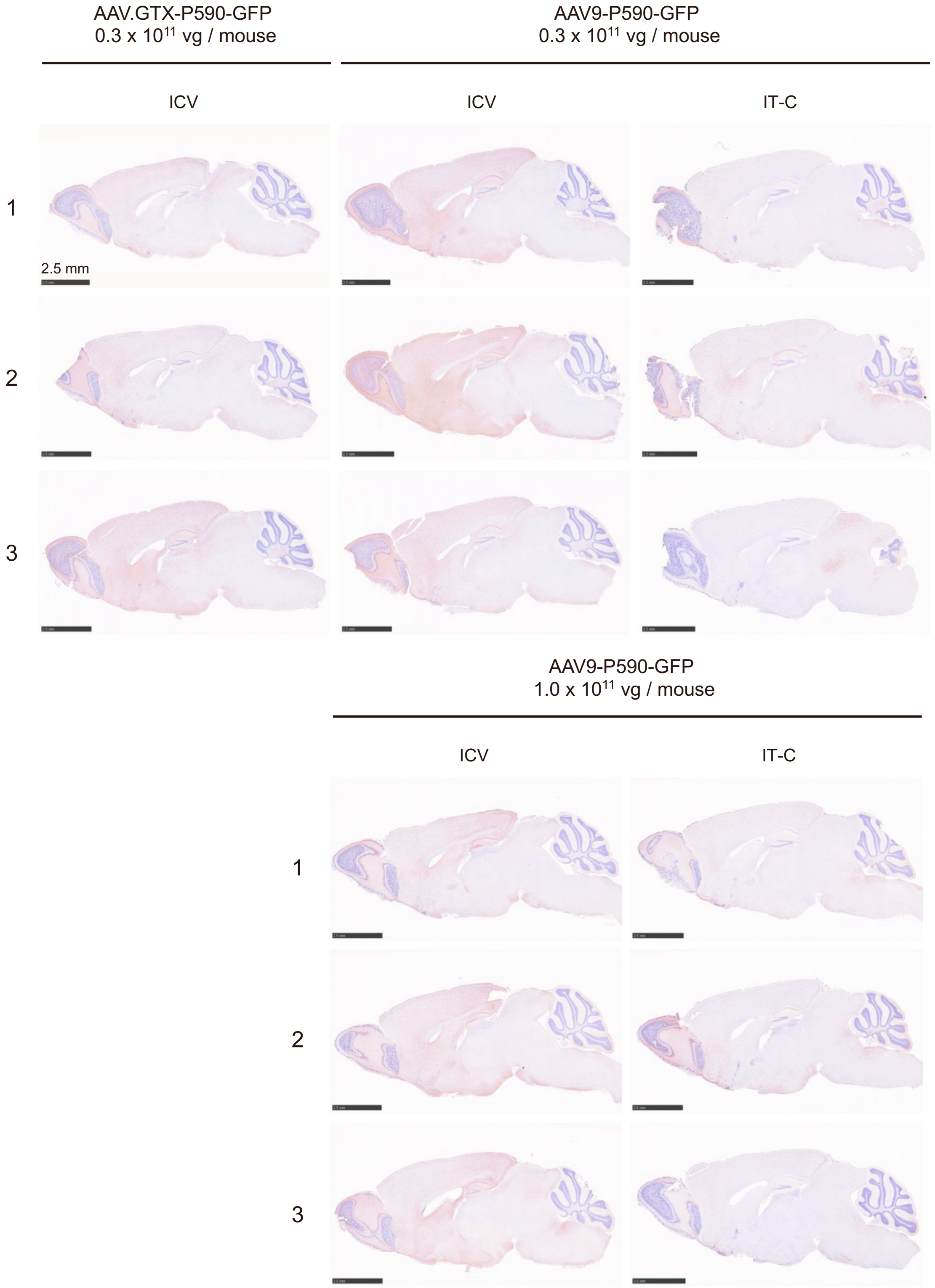

Figure S6. Comparison of DAB staining for GFP in tissue following ICV and IT-C administration of AAV.GTX-*P590-GFP* and AAV9-*P590-GFP* (0.3 or 1.0 × 10^{11} vg/mouse)

The photo shows the whole brain. Results of three cases in each group are shown.

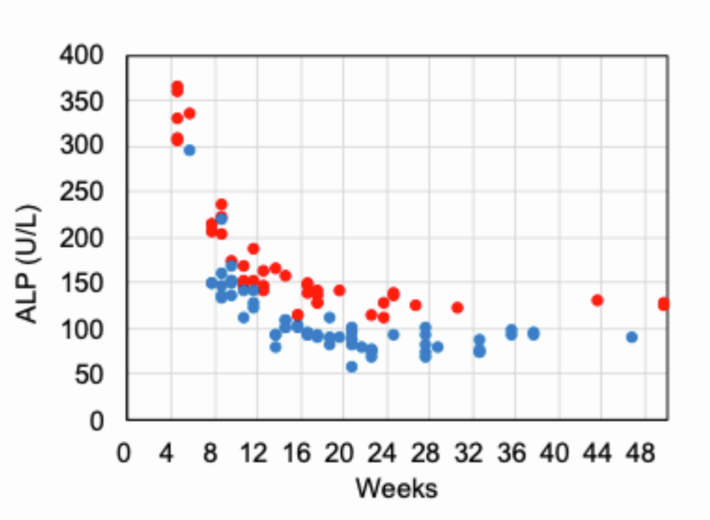

Figure S7. Age-related changes in plasma ALP levels in C57BL/6J mice

Blood samples were collected at various weeks after birth and ALP activity was measured separately for males and females. ALP levels were high in both sexes shortly after birth and gradually decreased by 16 weeks. Females tended to show higher levels than males. Based on these results, we decided to collect blood samples from 16 weeks onwards. Dots represent individual measurements; blue dots indicate males, red dots indicate females.

Video S1. Tremor in AAV-treated and untreated KI/KO mice

Untreated mice showed severe tremor, while AAV-treated mice appeared normal. Single black line on the tail indicates an AAV-treated mouse, two lines indicate an untreated mouse, and no line indicates a wild-type mouse.
